# A Dual Enrichment Strategy Provides Soil- and Digestate-Competent Nitrous Oxide-Respiring Bacteria for Mitigating Climate Forcing in Agriculture

**DOI:** 10.1128/mbio.00788-22

**Published:** 2022-05-31

**Authors:** Kjell Rune Jonassen, Ida Ormåsen, Clara Duffner, Torgeir R. Hvidsten, Lars R. Bakken, Silas H. W. Vick

**Affiliations:** a Faculty of Chemistry, Biotechnology, and Food Science, Norwegian University of Life Sciences, Ås, Norway; b VEAS WWTP, Slemmestad, Norway; c Helmholtz Zentrum München, Deutsches Forschungszentrum für Gesundheit und Umwelt (GmbH), Munich, Germany; CEH-Oxford

**Keywords:** agriculture, climate change, denitrification, microbial ecology, nitrous oxide, soil microbiology

## Abstract

Manipulating soil metabolism through heavy inoculation with microbes is feasible if organic wastes can be utilized as the substrate for growth and vector as a fertilizer. This, however, requires organisms active in both digestate and soil (generalists). Here, we present a dual enrichment strategy to enrich and isolate such generalists among N_2_O-respiring bacteria (NRB) in soil and digestates, to be used as an inoculum for strengthening the N_2_O-reduction capacity of soils. The enrichment strategy utilizes sequential batch enrichment cultures alternating between sterilized digestate and soil as substrates, with each batch initiated with limited O_2_ and unlimited N_2_O. The cultures were monitored for gas kinetics and community composition. As predicted by a Lotka-Volterra competition model, cluster analysis identified generalist operational taxonomic units (OTUs) which became dominant, digestate/soil-specialists which did not, and a majority that were gradually diluted out. We isolated several NRBs circumscribed by generalist OTUs. Their denitrification genes and phenotypes predicted a variable capacity to act as N_2_O-sinks, while all genomes predicted broad catabolic capacity. The latter contrasts with previous attempts to enrich NRB by anaerobic incubation of unsterilized digestate only, which selected for organisms with a catabolic capacity limited to fermentation products. The two isolates with the most promising characteristics as N_2_O sinks were *a* Pseudomonas sp. with a full-fledged denitrification-pathway and a *Cloacibacterium* sp. carrying only N_2_O reductase (clade II), and soil experiments confirmed their capacity to reduce N_2_O-emissions from soil. The successful enrichment of NRB with broad catabolic spectra suggests that the concept of dual enrichment should also be applicable for enrichment of generalists with traits other than N_2_O reduction.

## INTRODUCTION

The N_2_O concentration in the atmosphere is increasing, largely driven by the input of reactive nitrogen species in agriculture (1, 2). N_2_O emissions from farmed soils account for 52% of the total anthropogenic emissions of N_2_O (3) and approximately 1/3 of the climate forcing from food production (4). Limiting the input of reactive nitrogen to soils would be an effective mitigation measure but at the expense of lowering crop yields. This dichotomy has proven difficult to bypass, and estimates indicate only modest N_2_O mitigation potentials if currently available N_2_O abatement options were to be implemented at a large scale (5).

In agricultural soils nitrification and denitrification provide the main sources of N_2_O (6). Nitrous oxide reductase (Nos) is the only known enzyme catalyzing the reduction of N_2_O. Nos is expressed as part of the denitrification pathway sustaining anaerobic respiration by stepwise reduction of NO_3–_→NO_2–_→NO→N_2_O→N_2_, catalyzed by the enzymes nitrate reductase (Nar), nitrite reductase (Nir), nitric oxide reductase (Nor), and nitrous oxide reductase (Nos) encoded by the genes *nar*/*nap*, *nir*, *nor*, and *nosZ*, respectively (7). A significant share of denitrifying prokaryotes, however, are truncated, i.e., lacking genes encoding 1 to 3 of the four enzymes (8, 9), and truncated denitrifying pathways may significantly affect the N_2_O emissions in soils under denitrifying conditions. Organisms that lack all denitrification genes other than *nosZ* are particularly interesting, as they can act as net sinks for N_2_O. The propensity of the soil community to emit N_2_O can be reduced by increasing the relative abundance of such N_2_O-respiring bacteria (NRB) (10–12). However, as a stand-alone operation, such modification of soil microflora by inoculation would be prohibitively expensive.

We have previously demonstrated that anaerobic digestion (AD) provides a promising industrial platform for low-cost and large-scale introduction of N_2_O-reducing bacteria to soil (13). In that study, we enriched N_2_O-reducing bacteria in an unsterilized digestate by anaerobic incubation and isolated denitrifying bacteria with a strong preference for N_2_O over NO_3_, which could be grown aerobically to high cell densities in a sterilized digestate, providing an inexpensive inoculum for reducing N_2_O emission from soil. The isolated organisms did not include nondenitrifying NRB (bacteria with only *nosZ*), however, and it was evident that the catabolic capacity of the dominating organism was streamlined to harvest metabolic intermediates of the anaerobic consortium of the digestate and thus was poorly adapted for activity and survival in soil. Here, we present a new approach to obtain more suitable isolates through a deliberate attempt to enrich, and isolate, organisms that can grow in both digestate and soil. Conceptually the N_2_O-reducing organisms within a community can be divided into three categories according to their ability to grow/survive in digestate and soil: digestate specialists (D) with a competitive advantage in digestate, soil specialists (S) with a competitive advantage in soil, and generalists (G) capable of growth in both environments but plausibly at lower growth rates in both substrates relative to the two specialists. We hypothesized that we could enrich such generalists by sequential enrichment culturing, alternating between soil and digestate as the substrate (coined dual enrichment), and explored this with a Lotka-Volterra logistic growth model for the competition between three organisms, assigning hypothetical growth and death rates. The model revealed that alternating substrates could be used to enrich a reasonably competitive generalist after just a limited number of substrate transfers, even when the generalist organism began at a low abundance relative to its specialist competitors in the starting community.

**FIG 1:**
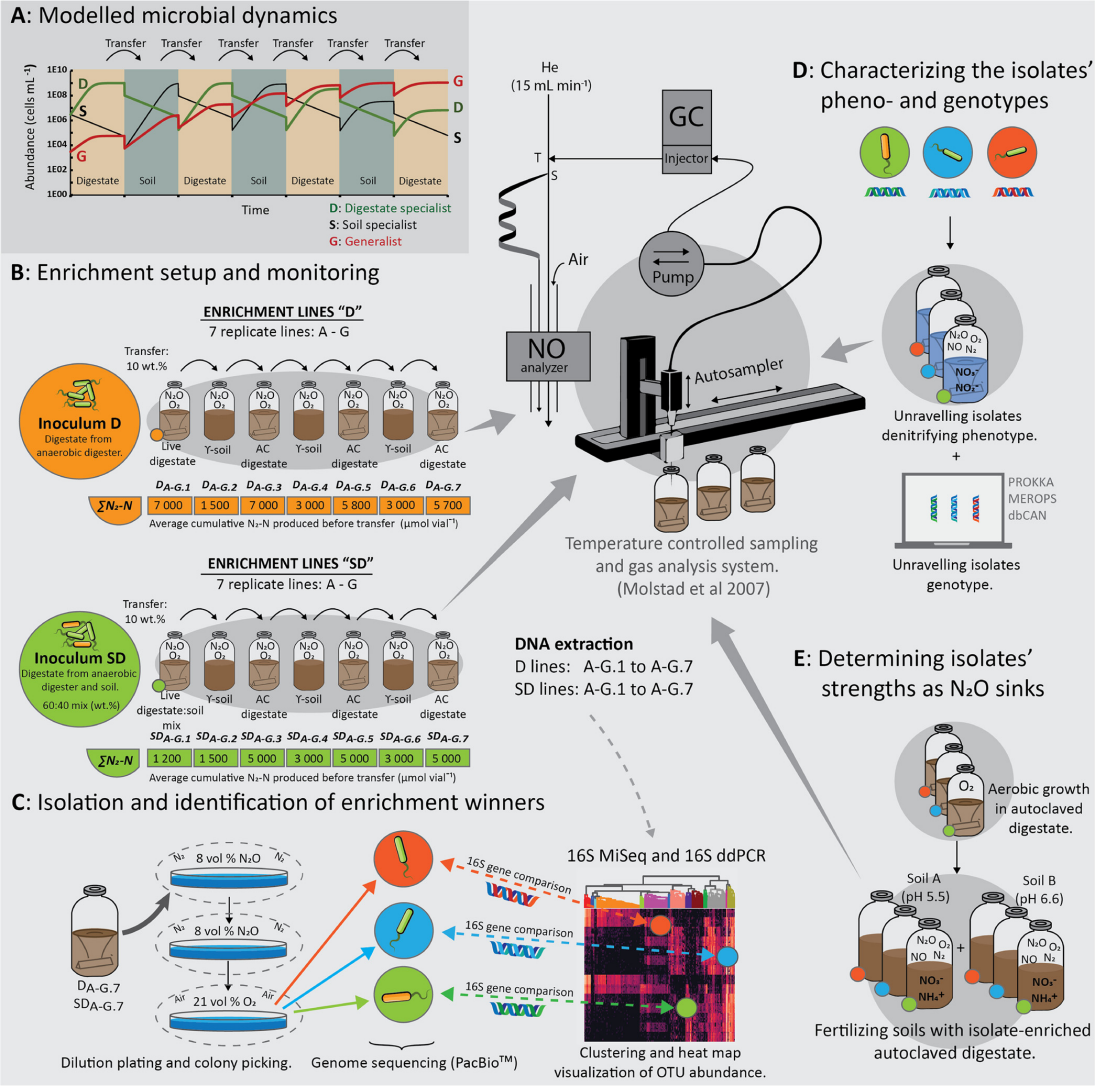
Graphical summary of materials and methods. (A) The dual enrichment was modeled by a set of Lotka-Volterra logistic equations for three organisms, digestate specialist (D), soil specialist (S), and generalist (G), competing for a common substrate pool. The repeated transfer of enriched material from one enrichment to the next, alternating between soil and digestate, was predicted to enrich generalists by nature. The modeling is presented in detail in Text S1. (B) Enrichment culturing experimental setup for the two enrichment lines, D (digestate-derived inoculum) and SD (soil- and digestate-derived mixed inoculum), each consisting of seven parallel replicate lines (A to G) over seven transfers. Each batch was supplemented with O_2_ and N_2_O (He background) in the headspace and monitored for O_2_, N_2_O, and N_2_ kinetics by frequent sampling of the headspace. While O_2_ was allowed to be depleted by respiration, N_2_O was sustained throughout by repeated injections. The average cumulative N_2_ produced for each culture is indicated below the vials (∑N_2_-N). DNA was extracted from every vial at the conclusion of each enrichment. (C) Extracted DNA was subjected to 16S rRNA gene amplicon sequencing, OTU clustering, and taxonomic assignment. The abundance of organisms circumscribed by each OTU was calculated from their relative abundance and the abundance of 16S rRNA gene copies mL^−1^ as measured with digital droplet PCR (ddPCR). The relative abundance of the 500 most abundant OTUs throughout the enrichment was clustered using the Ward variance minimization algorithm. This allowed for identification of clades of OTUs with similar development throughout the dual enrichments. The OTU’s 16S rRNA gene consensus sequences were aligned and matched with the 16S rRNA genes recovered from full-genome sequencing of axenic N_2_O-reducing isolates obtained from the final enrichments. (D) The isolates’ denitrifying phenotypes were assessed in pure culture incubations supplemented with either NO_3–_ or NO_2–_, and O_2_ or N_2_O and O_2_, and their phenotype was matched against their denitrifying genotypes. Eco-physiological genome analysis by annotation of carbohydrate-active enzymes, peptidases, denitrification reductase genes, and other genes provided insight into the suitability of these isolates as N_2_O-reducing inoculants for soil inoculation. (E) Each isolate was grown aerobically to high cell densities in autoclaved aerated digestate before amendment in two live soils (soil A: pH 5.5 and soil B: pH 6.6) supplemented with O_2_ and NO_3–_ to assess performance as N_2_O-reducing inoculants in soil.

Using this theoretical framework, we designed an enrichment strategy whereby a microbial community, originating from digestate or soil, was passaged through a series of batch enrichment cultures alternating between gamma-sterilized soil (γ-soil) and autoclave-sterilized digestate (AC-digestate) (Fig. 1). We anticipated that generalists would gradually increase in abundance throughout the enrichment series and that organisms that are noncompetitive in either substrate would be washed out due to the repeated dilution each transfer represents. Strong specialists would likely reappear when reintroduced in their preferred environment and thus be easily identifiable.

10.1128/mbio.00788-22.1 Text S1 Dual enrichment, conceptual model. Download Text S1, DOCX file, 0.4 MB. Copyright © 2022 Jonassen et al. 2022 Jonassen et al. https://creativecommons.org/licenses/by/4.0/ This content is distributed under the terms of the Creative Commons Attribution 4.0 International license.

In addition to this switching between the two substrates, we introduced an initial oxic/hypoxic phase in each enrichment culture by injecting O_2_ (which was allowed to be depleted by aerobic respiration), primarily to suppress obligate anaerobic organisms, but conceptually it would add another selective pressure, favoring organisms that tolerate rapid changes between oxia and anoxia. By means of this novel enrichment strategy, along with targeted isolation of N_2_O-respiring isolates, genome sequencing, and physiological experiments designed to unravel the isolates’ denitrifying regulatory phenotypes, we provide insight into the targeted enrichment of generalist-type NRB organisms and their performance as N_2_O-mitigating inoculants when vectored by digestate to agricultural soil.

## RESULTS AND DISCUSSION

### Growth modeling.

The Lotka-Volterra logistic growth model (explained in detail in Text S1 in the supplemental material) revealed that the selective pressure could be modulated by the duration of each enrichment and the fraction of enriched material transferred from one enrichment culture to the next, and predicted that a reasonably competitive generalist, having a growth rate of ≥50% of the growth rates of the two specialists in their respective substrates, would reach dominance after 7 repeated passages (Fig. 1), even when its initial abundance was 10^4^ times lower than the abundance of the two specialists. It also showed, however, that (i) an exceedingly high number of transfers was needed to secure dominance of a generalist with growth rates of <40% of the growth rates of the specialists and (ii) a generalist with a growth rate of <26% of that of the specialists would be eliminated.

### Dual enrichment culturing.

To enrich and isolate N_2_O-respiring organisms capable of strong growth in both digestate and soil, a dual enrichment approach was undertaken. Subjecting the enrichments to recurrent changes (i.e., growth substrate, oxic/anoxic) selects for organisms with a capacity to adapt rapidly to changing environmental conditions (14), a desirable trait for an organism destined for soil amendment.

Of note, the use of gamma-sterilized soil implies a selection of traits that enable organisms to tolerate abiotic soil factors, but not necessarily traits that enable organisms to withstand suppressive effects of the indigenous soil microbiota in live aerobic soil (15). While the inoculant communities for the enrichments contained all organisms present in the soil, which is likely to provide some level of biotic suppression/competition, this is gradually diluted out, as only N_2_O-reducing bacteria are selected for. The use of a sterilized soil material was necessary, however, to facilitate the enrichment of organisms.

**FIG 2:**
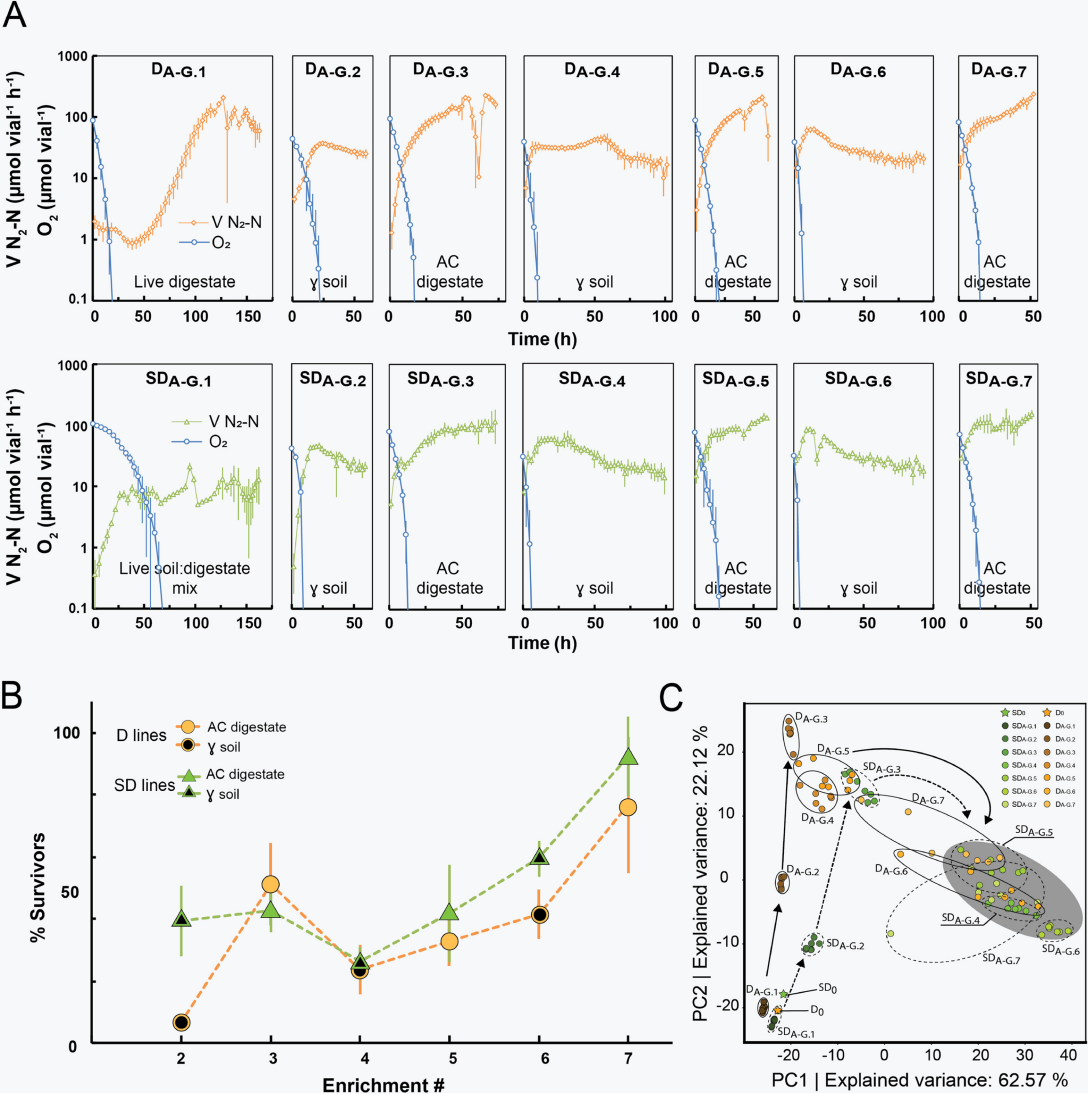
Gas kinetics and PCA of enrichment cultures. (A) Average rate of N_2_-N production for the two lines of enrichment culturing. A total of 10 weight percent of enriched material was transferred from one replicate vial to the next (D_A-G.j_ and SD_A-G.j_ to D_A-G_._j+1_ and SD_A-G.j+1_). AC digestate, autoclaved; γ-soil, gamma-sterilized. (B) Assessment of the fraction of the N_2_O-respiring community surviving transfer to the next enrichment cycle (details in Text S3C). (C) PCA of OTU relative abundances. Each dot represents an individual replicate (A to G). Standard deviation (*n* = 7) is shown as vertical bars (panels A and B).

The kinetics of N_2_O reduction to N_2_ throughout the consecutive enrichments is shown in Fig. 2A (more detailed analyses of the gas kinetics are shown in Text S3A and B). In the line D enrichment, inoculated with a live digestate community (D_A-G.1_), the N_2_-kinetics indicated the presence of two populations of N_2_O-respiring organisms, one whose activity was gradually declining, indicated by the log-linear decline of the N_2_ production rate (μ = −0.03 h^−1^) and a second population growing from initially extremely low numbers until their N_2_O respiration exceeded that of the declining population, increasing exponentially with a rate of 0.1 h^−1^ (modeled in Text 3A, top right panel). In contrast, the SD line enrichment, inoculated with a mixture of live soil and live digestate (SD_A-G_._1_), showed exponentially increasing rates for N_2_ production initially. Interestingly, the rates of N_2_-production in SD_A-G_._1_ did not reach as high as those of D_A-G.1_ (~10 versus ~120 μmol N_2_-N h^−1^ vial^−1^), which could be taken to suggest that (i) the N_2_O-reducing organisms originating from the soil quickly reached dominance due to the high initial numbers, (ii) these were less capable of scavenging electron donors in the digestate than the organisms originating from the digestate itself, and/or (iii) the indigenous digestate bacteria were suppressed by the soil bacteria. Throughout the subsequent enrichments, the N_2_ kinetics of the SD and D line became more similar, characterized by a short exponentially increasing rate and subsequent more or less stable rates. The seven-replicate series within each line (D and SD) had remarkably similar kinetics, reflected in the marginal standard deviation (Fig. 2A).

10.1128/mbio.00788-22.3 Text S3 Gas kinetics during enrichment culturing. Download Text S3, DOCX file, 0.3 MB. Copyright © 2022 Jonassen et al. 2022 Jonassen et al. https://creativecommons.org/licenses/by/4.0/ This content is distributed under the terms of the Creative Commons Attribution 4.0 International license.

In theory (see Text S1), the dual enrichment culturing should select for organisms that are able to grow both in soil and digestate (generalists, G) over the organisms that can only grow in soil (soil specialists, S) or digestate (digestate specialists, D), leading to a gradual increase in the G/(S+D) abundance ratio, which means that the percentage of N_2_O-respiring cells that survive the transfer to a new substrate (from soil to digestate and vice versa) should increase. We achieved crude estimates of the percentage of survivors for each transfer, based on the cumulated N_2_ in each enrichment and the initial rates in the next (explained in detail in Text 3C), and the results (Fig. 2B) lend support to the theory.

### Microbial community development in enrichment cultures.

The microbial community dynamics were analyzed based on 16S rRNA gene amplicon sequencing and operational taxonomic unit (OTU) clustering. PCA of community profiles demonstrated close similarity between replicate vials (A to G) throughout the first three enrichments and some divergence thereafter (Fig. 2C). Similarity percentage (SIMPER) analysis revealed that 10 OTUs accounted for 94.4 and 93.5% of the explained variance in the D and SD line, respectively, of which 8 OTUs were shared between the two lines (Text 4A and B). The D and SD lines followed similar trajectories and clustered in proximity to each other from enrichment SD_4_ and D_6_ forward, indicating a convergence toward a similar community structure (gray circle in Fig. 2C). The principal component analysis (PCA) clearly verified that the community underwent continuous dynamic succession and, surprisingly, that a high fraction of dominant OTUs were shared between the two lines.

By targeted isolation of N_2_O-respiring bacteria from the final enrichment cycle in autoclaved digestate (D_A-G.7_ and SD_A-G.7_), we obtained seven axenic N_2_O-respiring cultures and sequenced the genomes of six (Text S5A). The isolates were named according to genera with which they clustered in the phylogenetic tree generated with the 16S rRNA gene sequences of the isolates and related strains (Text S5C) and given working names: Pseudomonas sp. PS-02, *Aeromonas* sp. AM, *Brachymonas* sp. BM, *Ochrobactrum* sp. OB, *Cloacibacterium* sp. CB-01, *Cloacibacterium* sp. CB-03, and *Azonexus* sp. AN. AN was not genome sequenced, as its 16S rRNA gene partial sequence (obtained from Sanger sequencing of 16S rRNA gene PCR amplicons using 27F/1492R primer pairs) matched the 16S rRNA gene (99.2% sequence identity) of the dominating N_2_O-reducing *Azonexus* sp. (ERR4842639) isolated and characterized in the aforementioned experiments of Jonassen et al. (13).

10.1128/mbio.00788-22.4 Text S4 SIMPER analysis on 16S rRNA gene amplicon data. Download Text S4, DOCX file, 0.02 MB. Copyright © 2022 Jonassen et al. 2022 Jonassen et al. https://creativecommons.org/licenses/by/4.0/ This content is distributed under the terms of the Creative Commons Attribution 4.0 International license.

10.1128/mbio.00788-22.5 Text S5 Genome sequencing, phylogeny, and eco-physiological genome analysis of isolated organisms. Download Text S5, DOCX file, 0.4 MB. Copyright © 2022 Jonassen et al. 2022 Jonassen et al. https://creativecommons.org/licenses/by/4.0/ This content is distributed under the terms of the Creative Commons Attribution 4.0 International license.

The 16S rRNA genes recovered from the annotated genomes were compared to the representative 16S rRNA gene sequences of the OTUs to match isolates to OTUs from the 16S rRNA gene amplicon sequencing data. The isolates CB-01, CB-03, AN, AM, BM, and PS-02 were circumscribed by OTU1, OTU1, OTU2, OTU19, OTU37, and OTU8, respectively. These OTUs represented four of the top six most abundant OTUs of the D_A-G.7_ and SD_A-G.7_ samples. Including OTU74, circumscribing the isolate OB, five of the top 15 OTUs circumscribed the isolates. In summary, the average abundances of these OTUs were 59.8 ± 1.2% and 60.0 ± 1.1% in the D_A-G.7_ and SD_A-G.7_ enrichments, of which the dominating OTU1 accounted for 33% ± 10% and 39% ± 10% of the total abundance, respectively.

**FIG 3:**
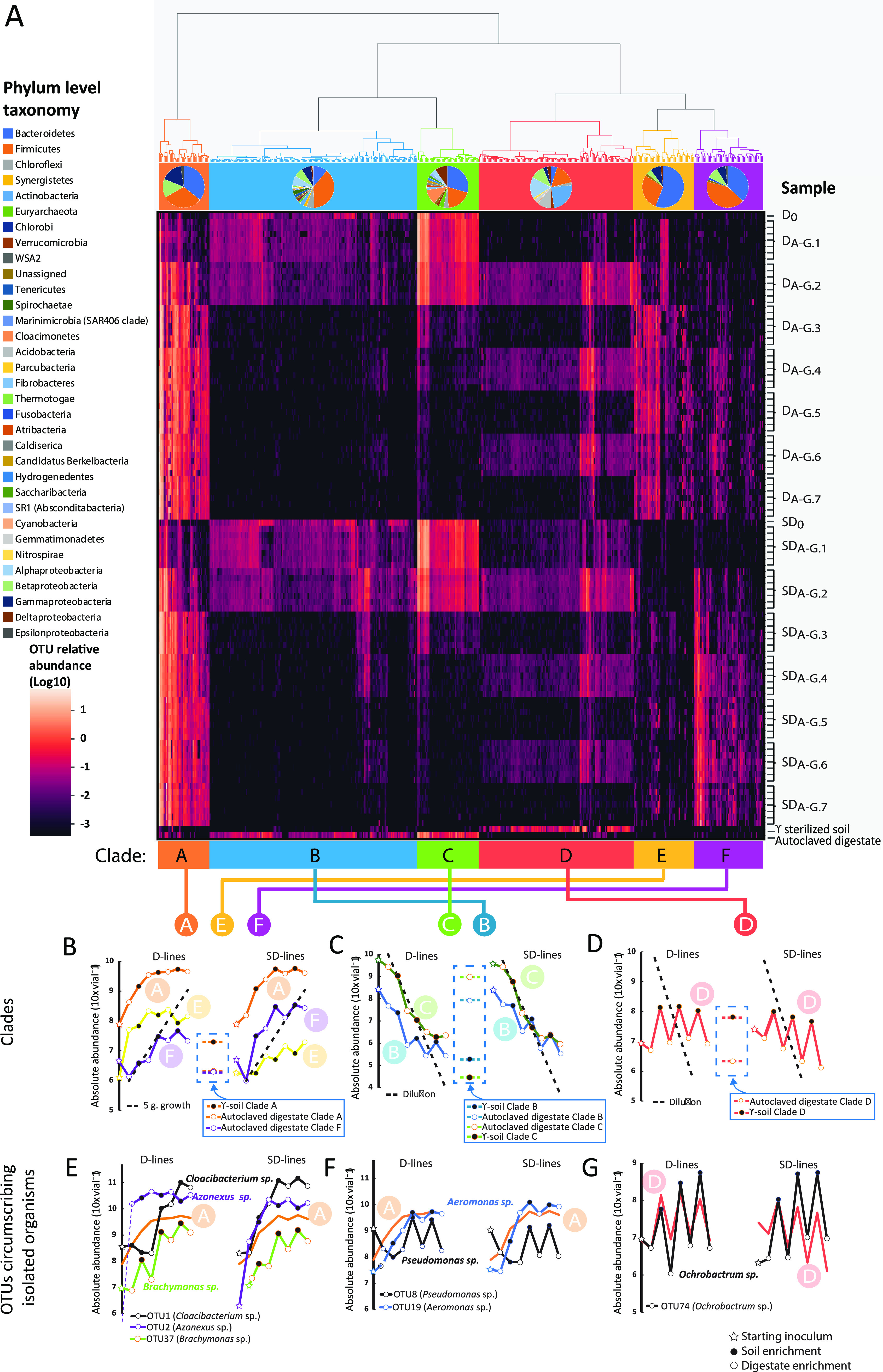
Abundance of clustered OTUs throughout the dual enrichment culturing. (A) Heatmapping and hierarchical clustering of the 500 most abundant OTUs from all biological replicates of the D line and SD lines of the dual enrichment culturing, including starting inocula (D_0_ and SD_0_) and background samples of γ-soil and autoclaved digestate used as growth medium in the enrichments. OTUs are arranged in columns and samples in rows. The clustering has been delineated into six clades (A, B, C, D, E, F), with phylogenetic composition of OTUs in clades displayed below the cladogram. (B to D) The average absolute abundances (copies vial^−1^) for the OTUs within each clade throughout each enrichment; filled symbols, enrichment in soil; open symbols, enrichment in digestate; star, starting inoculum. The dashed lines in panels C and D represent the predicted decline by dilution, given a 10% transfer rate, i.e., neither growth nor death. The dashed line in panel B represents a growth rate of 5 generations per enrichment. The OTU abundances in sterile materials are shown within dashed frames. Panels E to G show the abundance of the OTUs which circumscribe the isolates, together with the averages of their resident clades.

The dynamic change in OTU abundance of the 500 most abundant OTUs (sum abundance across all samples) throughout the consecutive enrichments of the D and SD lines was hierarchically clustered based on Euclidian distance measures and visualized by heatmapping of OTU relative abundance (Fig. 3A). The hierarchical clustering identified six clades, denoted A to E in Fig. 3A, that clustered OTUs according to their abundance patterns throughout the consecutive enrichments. To achieve a more quantitative assessment of the phenomena portrayed in the heatmap, the absolute abundances were estimated by combining the total 16S rRNA gene abundance (Text 5B) with the relative abundance of each clade and individual OTU (Fig. 3B to D). This analysis included an assessment of the relative increase of individual OTUs in each enrichment culture (*R_i_*). The average *R_i_* for soil (*R_Soil_*) and for digestate enrichments (*R_Digestate_*) for each OTU was used to judge whether the OTU is a soil specialist (high *R_Soil_*, low/negative *R_Digestate_*), a generalist (high *R_Soil_* and *R_Digestate_*), or a digestate specialist (high *R_Digestate_*, low/negative *R_Soil_*).

Most OTUs within clade A were present initially in both enrichment lines (D_0_ and SD_0_), suggesting a primarily digestate origin of these OTUs, of which most were assigned to the phyla *Bacteriodetes*, *Cloacimonetes*, and *Betaproteobacteria* (Fig. 3A). Clade A showed an increase in abundance throughout the enrichment in both enrichment lines (Fig. 3B) with an increase equivalent to ~5 cell divisions in the first 3 to 4 enrichment cultures (dashed line, Fig. 3B). Inspection of the growth of individual OTUs (*R_i_* values) within clade A showed that they were able to grow both in digestate and soil but spanned a range from soil specialists (*R_Digestate_* close to zero) to generalists (R_Soil_ and R_Digestate_ >2; Text S6A). The OTUs circumscribing the isolated cultures CB-01 (OTU1), CB-03 (OTU1), AN (OTU2), PS-02 (OTU8), AM (OTU19), and BM (OTU37) were all within clade A (Fig. 3E and F). OTU2, circumscribing *Azonexus* sp. AN, grew better in digestate than in soil (*R_Digestate_* 3.40 ± 0.35 and R_Soil_ 2.27 ± 0.35) and reached dominance in the first enrichment in live digestate (D_A-G.1_ culture vials), which was also observed in the enrichments of Jonassen et al. (13).

10.1128/mbio.00788-22.6 Text S6 Assessment of growth or death of OTUs within the dual substrate enrichment. Download Text S6, DOCX file, 0.2 MB. Copyright © 2022 Jonassen et al. 2022 Jonassen et al. https://creativecommons.org/licenses/by/4.0/ This content is distributed under the terms of the Creative Commons Attribution 4.0 International license.

Clades B and C plausibly harbored digestate-derived OTUs, which were diluted out, rather than dying out, since their abundance declined with a rate largely as predicted by the dilution rate (Fig. 3C and Text S6B and C). In autoclaved digestate, the absolute abundance of OTUs clustered in clades B and C was ~10^8^ and 10^9^ vial^−1^, respectively, while the abundance at the end of each enrichment was much lower, suggesting that their DNA is not destroyed by autoclaving, but that this relic DNA is degraded once the digestate is inoculated with live organisms. Thus, the high degree of clustering of samples by PCA (Fig. 2C) in the initial enrichments is probably not influenced by relic DNA as reported by others (16).

Clade D appeared to consist of soil specialists that sustain abundance in soil only, or alternatively, are partly made up of relic DNA (DNA in the γ-sterilized soil) not metabolized during the enrichments in soil, as mineral or humic substances may protect free DNA from rapid degradation (17). However, some did appear to be true soil specialists due to their absence in the γ-sterilized soil (Fig. 3A). Our quantitative assessment confirmed that clade D organisms grew in soil but declined in digestate (Fig. 3D; see calculated *R* values in Text S6D). This clade harbored the soil specialist OTU74, circumscribing the isolated *Ochrobactrum* sp. OB (Fig. 3G), demonstrating the predicted characteristics of a soil specialist, and reappearing at high abundance in soil enrichments.

Clade E showed an average increase in abundance throughout the enrichment in both enrichment lines and appeared to be able to grow in both soil and digestate (Text S6E). Interestingly, clade E harbored organisms enriched to higher levels in the digestate-derived line (D line) compared to the SD line (Fig. 3B), suggesting that they were suppressed by some organisms originating from the soil. Clade F appeared to contain many organisms that grew better in soil than in digestate (Text S6F) and that were enriched in the SD line to a greater degree than in the D line (Fig. 3). Some of the OTUs in this clade appear to be soil-derived organisms, and for some, their abundance in the D line could be overinflated by the presence of relic DNA (from the γ-soil) (17) or due to an artifact of sequence OTU clustering.

### Eco-physiology of the isolated organisms as inferred from genome analyses.

In the enrichment cultures, the N_2_O reduction rates during the batch cultures suggested that the growth of the N_2_O-respiring organisms was C-substrate-limited most of the time (Fig. 2A). Tracing the OTUs circumscribing the isolated organisms throughout the enrichment cycles showed that many of these organisms grew to, and maintained, high abundances throughout the repeated transfers, i.e., growing in both materials (Fig. 3E to G). Acquisition of less accessible C-substrates could therefore in part explain why the isolated organisms outperformed other species throughout the enrichments. To explore this metabolic utilization of less accessible C-sources, we examined the isolate genomes in the context of carbohydrate-active enzyme (CAZyme) and peptidase genes (Data Set S1).

10.1128/mbio.00788-22.9 Data Set S1 dbCAN annotations of carbohydrate-active enzymes in isolate genomes. Download Data Set S1, XLSX file, 0.1 MB. Copyright © 2022 Jonassen et al. 2022 Jonassen et al. https://creativecommons.org/licenses/by/4.0/ This content is distributed under the terms of the Creative Commons Attribution 4.0 International license.

All isolates carry a range of CAZyme genes (Text S5D). Several of these, which are known to target complex carbohydrates, also contained putative signal peptides, indicating that these proteins are transported to the cell exterior and may be used for the extracellular degradation of complex carbohydrates. Isolates CB-01 and CB-03 seemed to have CAZymes focused on the breakdown of plant materials, coding enzymes involved in binding and degradation of cellulose, cellulose derivatives, and starch (Text S5D and E). AM also had a large repertoire of genes encoding CAZymes with multiple carbohydrate binding modules (CBMs) associated with cellulose (CBM5) (18), starch/glycogen (CBM48), peptidoglycans, and chitin binding (CBM50) (19) (Text S5D to E). Isolates also contained many genes involved in glycogen synthesis and breakdown, a trait which could provide a fitness advantage during dual culture enrichment, as glycogen metabolism has been shown to improve Escherichia coli fitness when experiencing changing environments (20). In contrast to the other isolates, BM did not appear to be geared toward extracellular degradation of complex carbohydrates, nor did it contain genes involved in glycogen metabolism (Text S5D and E).

While all isolates included peptidases containing putative signal sequences, the relative proportion of these varied between the isolates, with CB-03 having the largest proportion of predicted peptidases containing putative signal sequences, followed by AM and CB-01 (Text S5F). Interestingly, the peptidases seen in the isolate genomes were those active in the neutral pH range and not low-pH active peptidases (21). This falls in line with the pH of the environments from which the isolates were obtained, i.e., neutral/alkaline digestate and weakly acidic soil. Also of note, the ability of the isolates to grow in sterilized digestate (high *R_Digestate_*) was strongly correlated with the number of genes coding for proteases and CAZymes, suggesting that these enzyme classes drove successful colonization of the sterilized digestate environment (File S8A).

### Characterizing the isolates’ denitrifying regulatory phenotypes (DRP) and genotype.

**FIG 4:**
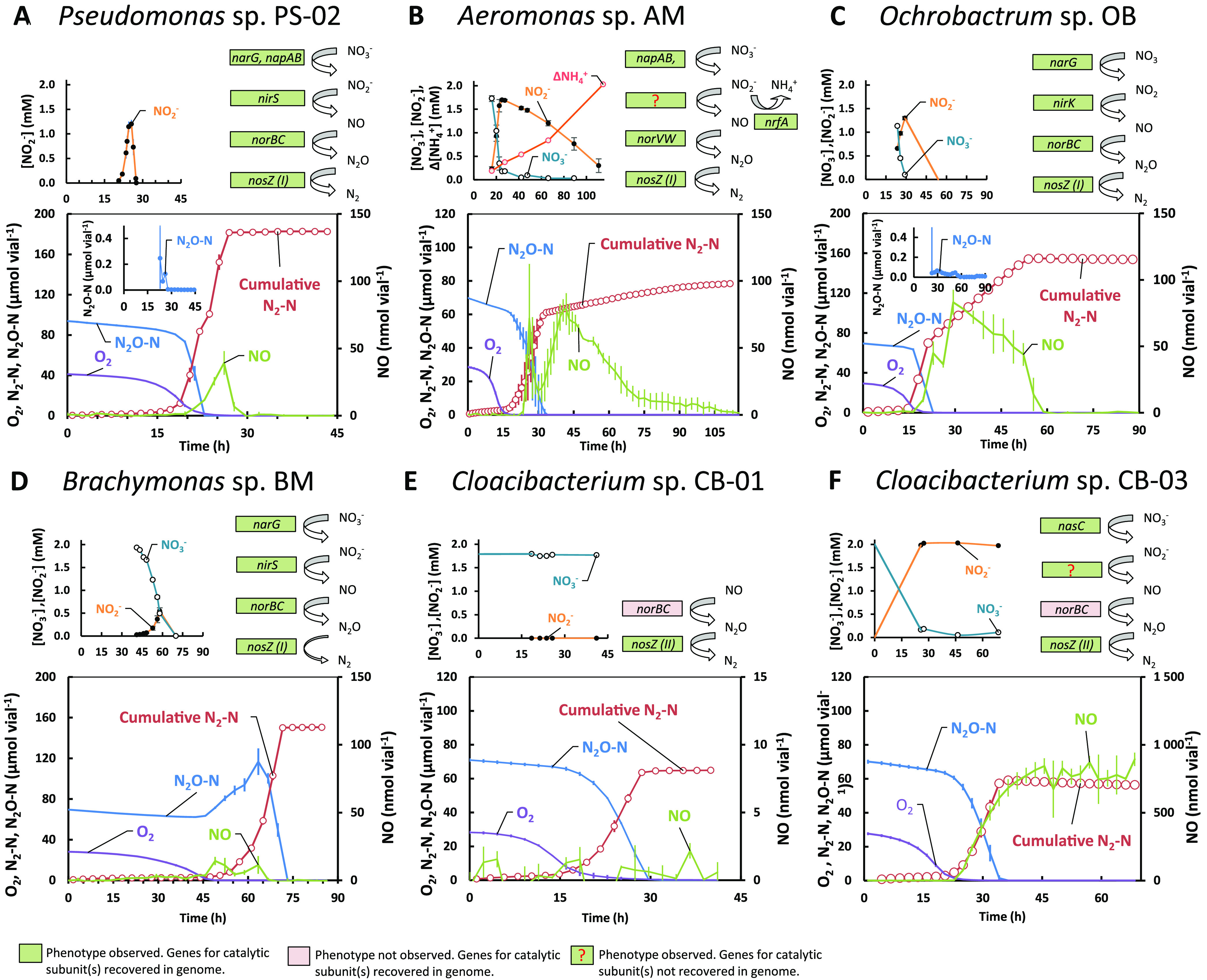
Denitrification genes and denitrification phenotypes of isolated organisms. Gas kinetics of O_2_, N_2_O-N, NO, and cumulative N_2_-N (adjusted for leakage and sampling) in denitrifying phenotype experiments in 120-mL closed vials with He atmosphere containing 50 mL liquid growth medium supplemented with 41.6 μmol O_2_, 41.6 μmol N_2_O, and 2 mM NO_3–_. Liquid concentrations of NO_2–_, NO_3–_, and/or NH_4_^+^ (small panels; dashed lines, estimated by N-mass balance), and all genes coding for catalytic subunits of N-reductases recovered from the Prokka annotated genomes. (A) Pseudomonas sp. PS-01 (*n* = 2) grown in SS medium. PS-02 demonstrated strict control of gaseous denitrification intermediates throughout the incubation. (B) *Aeromonas* sp. AM (*n* = 3) grown in SS medium. AM demonstrated a DNRA + NOS phenotype, converting NO_3–_ to NO_2–_ and NH_4_^+^. Denitrification was ongoing throughout but at a low and constant rate (0.4 μmol N_2_-N h^−1^ vial^−1^). (C) *Ochrobactrum* sp. OB (*n* = 2) grown in SS medium. OB demonstrated strict control of gaseous intermediates throughout the incubation. (D) *Brachymonas* sp. BM (*n* = 2) grown in Sistrom’s succinate medium. BM demonstrated a full-fledged denitrifying phenotype where N_2_O was kept at high levels throughout the incubation. (E) *Cloacibacterium* sp. CB-01 (*n* = 3) grown in NB medium. CB-01 had a truncated denitrifying phenotype respiring primarily N_2_O. (F) *Cloacibacterium* sp. CB-03 (*n* = 2) grown in NB medium. CB-03 had a truncated denitrifying phenotype converting N_2_O to N_2_ and nitrate to nitrite.

All isolates carried the gene *nosZ* (clade I or II (22), as well as several other denitrification genes (Fig. 4). Although organisms with a full-fledged denitrification pathway can both produce and reduce N_2_O, they may act as strong sinks for N_2_O in the environment, depending on their denitrification regulatory phenotype (DRP) (23), which is shaped by the regulatory network controlling the stepwise reactions of denitrification, at both the transcriptional (24) and metabolic (25) levels.

The denitrification regulatory phenotypes of the isolates were investigated by monitoring the kinetics of O_2_, NO, N_2_O, NO_2–_, and NO_3–_ in stirred batch cultures as they depleted the oxygen and switched to anaerobic respiration, as described in Jonassen et al. (13). Measured gases in incubations supplemented with 41.6 μmol O_2_, 41.6 μmol N_2_O, and 2 mM NO_3_-, along with measured liquid concentrations of NO_2–_, NO_3–_, and NH_4_^+^ and genes coding for catalytic subunits are shown for each isolate in Fig. 4.

The genomes of Pseudomonas sp. PS-02, *Ochrobactrum* sp. OB, and *Brachymonas* sp. BM predicted a full-fledged dentification pathway, i.e., reduction of NO_3–_ to N_2_, which was verified through phenotyping experiments (Fig. 4A, C, and D). However, the regulatory phenotypes were profoundly different: PS-02 reduced available NO_3–_ and N_2_O concomitantly, before initiating NO_2–_ reduction (Fig. 4A). Nos activity was higher than that of the other N reductases at the oxic/anoxic transition, as there was only miniscule, transient accumulation of N_2_O during denitrification, and the preferential reduction of N_2_O was maintained if cultured with NO_2–_, with or without N_2_O in the headspace. The phenotype of OB (Fig. 4C) was very similar to that of PS-02. BM, however, reduced most of the available NO_3–_ to N_2_O initially (Fig. 4D), a trait which was retained if cultured with NO_2–_, with or without N_2_O in the headspace. This suggested that while BM would be a source of N_2_O in the environment, PS-02 and OB could be strong sinks, provided these phenotypes occur in natural settings.

Dissimilatory nitrate reduction to ammonium (DNRA) organisms with *nosZ* could be attractive inoculants since they reduce NO_3–_ to NH_4_^+^ rather than to N_2_, thus retaining plant-available N in the soil (26) while at the same time scavenging N_2_O produced by other organisms. The AM isolate, possessing genes for a DNRA pathway, simultaneously reduced the available NO_3–_ to NO_2–_ and N_2_O to N_2_ after O_2_ depletion (Fig. 4B) and subsequently reduced NO_2–_ to NH_4_^+^ and trace amounts of N_2_. This indicated DNRA, which was corroborated by the presence of *nrfA* in the genome, coding for a key enzyme of DNRA (cytochrome *c*552 nitrite reductase, EC 1.7.2.2) (27). It also carried a *nasD* gene that showed high sequence similarity (protein blast) with NirB (NADH-dependent nitrite reductase) of a related *Aeromonas* strain. Genes for the nitrite reductases NirS/K were not identified, and the source for the produced NO remains unresolved. The AM genome also apparently lacked genes for the nitrate reductase NarGHI, while genes coding periplasmic nitrate reductase Nap (*napAB*) and N_2_O reductase Nos (*nosZ*, clade I) were present. It also possessed a gene annotated as *nasA*, coding a constituent of the nitrate assimilatory system (Nas) in a wide range of bacteria (28). The phenotypic analysis showed that NO_3–_ and N_2_O were clearly reduced at the same time in incubations with the AM isolate (Fig. 4B). This contrasts with earlier findings that Nos outcompetes Nap for electrons in denitrifying bacteria (25).

The genotypes of *Cloacibacterium* sp. CB-01 and CB-03 predicted a truncated denitrification pathway (NO→N_2_O→N_2_), and one (CB-03) was also equipped with genes for assimilatory NO_3–_ reductase (*NasC*, EC 1.7.99.4) and a nitrite/nitrate transporter (*narK*). This was verified by experiments showing stoichiometric conversion of N_2_O to N_2_ and reduction of NO_3–_ to NO_2–_ by CB-03 (Fig. 4E and F). Early onset of NO_3–_ reduction, before depletion of oxygen, suggested that NasC was active under oxic conditions in this isolate, which was also reported for Paracoccus denitrificans (29). Of the two isolates, CB-01 makes for a particularly promising N_2_O-reducing soil inoculant. Both CB-01 and CB-03 were circumscribed by OTU1 of clade A (Fig. 3E), which dominated both D and SD enrichment lines. Growth experiments where NO_3–_ was provided with and without high concentrations of exogenous N_2_O showed that the regulation and expression of denitrification genes were unaffected by N_2_O levels for all isolates.

### Performance of isolated organisms as sinks for N_2_O in soil.

To produce inocula for testing the isolates’ capacities as N_2_O sinks in soil, they were grown aerobically to high cell densities in autoclaved digestate (Text S8A). The estimated cell density at the end of the 45 h of incubation ranged from 0.5 to 1.4 mg dry weight mL^−1^ (~3 to 7 · 10^9^ cells mL^−1^) for the different isolates; the lowest value recorded was for *Brachymonas* sp. BM (0.5 mg dry weight mL^−1^), while *Aeromonas* sp. AM reached the highest (1.4 mg dry weight mL^−1^). Interestingly, the capacity of the isolates to grow to high density was strongly correlated with the number of genes coding for CAZymes and proteases in their genomes (Text S8A).

10.1128/mbio.00788-22.8 Text S8 Growth in autoclaved digestate and effects on soil emissions. Download Text S8, DOCX file, 1.8 MB. Copyright © 2022 Jonassen et al. 2022 Jonassen et al. https://creativecommons.org/licenses/by/4.0/ This content is distributed under the terms of the Creative Commons Attribution 4.0 International license.

**FIG 5:**
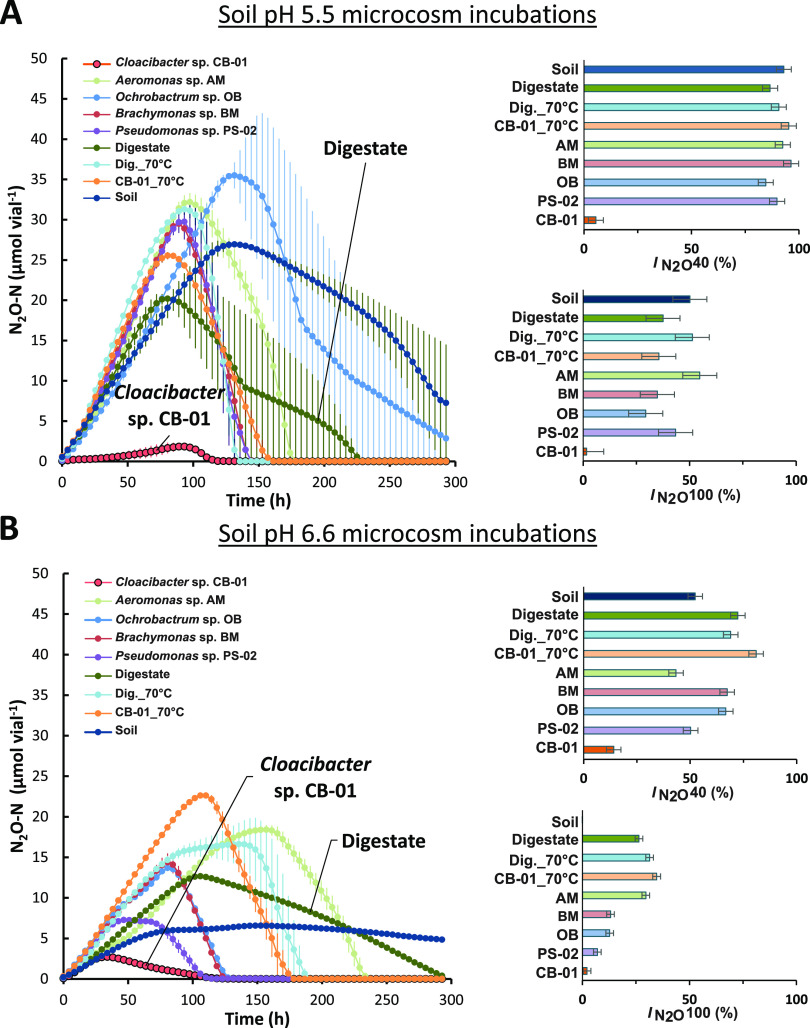
(A and B) Gas kinetics during incubation of soils (soil pH = 5.5 in panel A and soil pH = 6.6 and panel B) amended with various pretreated digestates (0.06 mL g^−1^ soil). The experiment included 4 control treatments without isolates; soil, soil without any amendment; digestate, digestate directly from the anaerobic digester; Dig_70°C, digestate heat treated to 70°C for 2 h CB-01_70°C, autoclaved digestate in which CB-01 was grown (as for the CB-01) treatment) and subsequently heated to 70°C for 2 h. The treatments with live isolates present included autoclaved digestate in which isolates AM, BM, OB, PS-02, and CB-01 had been grown aerobically to cell densities of 1.39, 0.51, 0.79, 0.81, and 0.72 mg cell dry weight mL^−1^, respectively (Text S8A). The main graphs (panels A and B) show N_2_O kinetics for each treatment (*n* = 2). The bar graphs to the right show the N_2_O indexes expressed as a percentage (average *I_N2O_* for each treatment, with 95% confidence intervals based on analysis of variance [ANOVA] and Tukey’s test). *I_N2O_* is the area under the N_2_O-curve as a percentage of area under the N_2_O+ N_2_ curve. Two *I_N2O_* values are shown for each soil: one for the timespan until 40% of the NO_3–_-N is recovered as N_2_ + N_2_O + NO-N (I_N2O 40%_) and one for 100% recovery (I_N2O 100%_). More details (including N_2_ and NO kinetics) are shown in Text S8B and C.

To assess the N_2_O sink capacity of these aerobically grown organisms, they were inoculated to soil in vials with He atmosphere (with traces of O_2_), which were monitored for O_2_, NO, N_2_O, and N_2_ during a 300-h incubation. For each treatment, we calculated the *I_N2O_* emission ratio, which is the area under the N_2_O curve divided by the area under the N_2_O+N_2_ curve (30), expressed as a percentage, which is a proxy for the propensity of N_2_O emissions from denitrification (31). Since the effect of the inoculation confounds the impact of the isolates because of the added available carbon introduced by the digestate, we included four control treatments (see legend of Fig. 5). The most relevant of these controls for assessing the N_2_O sink capacity of the isolates is “CB-01_70°C” since this digestate was identical to that with isolates present, except for the final heat treatment to kill CB-01.

As expected, *I_N2O_* values were generally higher in the pH 5.5 soil than in the pH 6.6 soil (Fig. 5), and the isolates BM, OB, and PS-02 lowered *I_N2O_* only in the soils with pH 6.6 (*P* < 0.025, for the contrast between bacterial treatments and the control treatment CB-01_70°C). In contrast to the other isolates, CB-01 resulted in extremely low *I_N2O_* values in both soils, clearly outperforming any of the control treatments. We tested if the ability of CB-01 to act as a strong N_2_O sink in the pH 5.5 soil could be due to acid tolerance by growing CB-01 in stirred (600 rpm) liquid medium with pH ranging from 5.5 to 7 and found no evidence for acid tolerance, either for growth or for the synthesis of functional N_2_O reductase (Text S7). An alternative explanation of the acid-tolerant N_2_O sink effect of CB-01 could be that the cells were embedded in flocks/biofilms in the digestate, protected against low soil pH by the buffer capacity of the matrix or attachment to more alkaline soil particles. Strains of *Cloacibacterium* are known to secrete extracellular polymeric substances (32) and are found in high abundance in biofilms of wetlands (33), which lends support to the hypothesis of matrix-mediated shielding effects. This points toward the advantages of biofilm formation or other attachment strategies in generating favorable micro niches and so gaining advantage over competitors in a low-pH environment.

10.1128/mbio.00788-22.7 Text S7 pH-dependent aerobic and anaerobic respiration by *Cloacibacterium* sp. CB-01. Download Text S7, DOCX file, 0.1 MB. Copyright © 2022 Jonassen et al. 2022 Jonassen et al. https://creativecommons.org/licenses/by/4.0/ This content is distributed under the terms of the Creative Commons Attribution 4.0 International license.

While our eco-physiological genome analysis revealed that several isolates had the genetic potential to utilize complex carbon sources and had several traits that might secure survival in a competitive situation, agricultural inoculants are most definitely invaders of the soil microbial community, and any longer-term establishment is dependent on the resistance by the residential community against alien species. The likelihood of a successful invasion is related to the resident community richness, referred to as the diversity-invasion effect (15) and reflects the key challenges of an invading organism—growth and establishment by utilizing resources not utilized by the resident community or forcefully “overtaking” a resident niche through competition or antagonism.

To assess the ability of our isolates to persist in soil and to retain their N_2_O reduction capacity, a second experiment was set up with identical treatments to those in Fig. 5 but storing the amended soils for 1 month with exposure to atmospheric oxygen before testing the denitrification kinetics. A fertilization event was simulated by the addition of 50 μmol NO_3–_, 1 mg ground plant material g^−1^ soil, and 20.8 μmol O_2_ before sealing vials and monitoring denitrification kinetics throughout depletion of oxygen and the transition to anoxia. In this experiment the effect of the inoculated isolates on N_2_O emissions was evaluated based on maximum N_2_O accumulation (no treatment reduced all available N oxides, making it impossible to calculate *I_N2O_* emission indexes) (Text S8D and E). While none of the inoculants significantly differed from the controls in pH 5.5 soil, PS-02 outperformed the other inoculants at pH 6.6. In fact, the soil treated with PS-02 performed better after 30 days of soil storage (maximum N_2_O for PS-02 was ~1/10 of other treatments; Text S8E) than immediately after amendment in the first soil experiment (Fig. 5). Likewise, maximum N_2_O for CB-01 treatment in pH 6.6 soil was approximately 2/3 that of other amendments, but the difference was not statistically significant (*P* > 0.05).

A dose-response experiment with the isolates CB-01, PS-02, and OB grown freshly in digestate was conducted to determine the minimum dose needed to obtain substantial reduction of N_2_O production in soil. The highest inoculation intensity in this experiment (6 · 10^7^ cells g^−1^ soil) is approximately 50% of that used in the previous experiments (Fig. 5).

The results, summarized in Text S8G and H, showed a strong dose-dependent effect of *Cloacibacterium* sp. CB-01 on N_2_O accumulation, exemplified with the peak N_2_O concentration (maximum [max] N_2_O), which was reduced by 96%, 70%, and 20% (compared to the control without bacteria) for the inoculation levels 0.6, 0.3, and 0.15 mL digestate vial^−1^, respectively (*P* < 0.025 for all contrasts). Pseudomonas sp. PS-02 and *Ochrobacter* sp. OB had weaker effects on max N_2_O, but statistically significant (*P* < 0.05) at all inoculation levels. The *I_N2O_* showed the same patterns, although some contrasts (isolates versus control) lacked statistical significance for the lowest inoculation dose.

Our inoculation levels were 2.7, 4.5, and 9 · 10^7^ cells g^−1^ soil, which is within the upper range of inoculation levels used by Domeignoz-Horta et al. (11), who inoculated soils with 10^6^ and 10^8^ Dyadobacter fermentans cells g^−1^ soil. Dyadobacter fermentans carries *nosZ* clade II, but no other denitrification genes, which makes it comparable to our *Cloacibacterium* sp. CB-01, and a comparison of the performance of the two isolates is interesting: inoculation with 10^8^ *D. fermentans* cells g^−1^ resulted in a reduction in the N_2_O/(N_2_O+N_2_) product ratio which is similar to what was achieved by the two highest inoculation levels with *Cloacibacterium*, i.e., 0.45 · 10^8^ to 0.9 · 10^8^ cells g^−1^. Thus, the two organisms appear to have similar capacities for acting as sinks for N_2_O in soil. However, inspection of Domeignoz-Horta et al. (11) reveals that *Dyadobacter* did not affect the N_2_O-emission in soils with pH below 6.6, while *Cloacibacterium* performed well in our acid soil (pH 5.5, Fig. 5A). This could indicate that *Cloacibacterium* sp. CB-01 has a more robust N_2_O sink capacity in low-pH soils. As suggested previously, this is probably not due to an inherent acid tolerance, but rather, to a combined effect of the organism’s tendency to aggregate and form biofilms and the relatively high pH of the digestate (pH 7.6). The matrix in which cells are embedded prior to inoculation to soils is probably a crucial issue.

### Concluding remarks.

The hierarchical clustering of 16S rRNA gene-based OTUs demonstrated that the dual enrichment effectively selected generalist organisms capable of growth by N_2_O respiration in both sterilized digestate and soil, after just 3 to 4 transitions, as predicted by the model (Text S1). Among the isolates, *Cloacibacterium* sp. CB-01 stands out as particularly interesting, as it grew well both in soil and digestates and was unable to denitrify *sensu stricto* (lacking the genes for dissimilatory NO_3–_ and NO_2–_ reduction). In addition, it proved a strong N_2_O sink even in the acidic soil (pH 5.5), where the other isolates’ synthesis of functional N_2_O reductase appeared to be hampered by low pH, as is the case for most organisms (30, 34). Testing the pH response of CB-01 in pure culture showed no particular tolerance to low pH in axenic liquid culture (Text S7), however. We speculate that CB-01’s ability to reduce N_2_O in low pH soil is due to the ability of this organism to localize in alkaline microniches supplied by the digestate material, possibly through the production of a biofilm, a trait known to be common to members of this genus (33, 35–37). The ability to reduce N_2_O in low-pH soils is very desirable in agricultural settings due to the issue of soil acidification, driven by N input and subsequent base cation depletion in agricultural soils (3), which enhance N_2_O emission (30). Liming such acidified soils would mitigate their N_2_O emissions (38, 39), but at the possible expense of increased emissions of carbonate-CO_2_ (40). The second isolate to show promise is PS-02. While PS-02 can act as both a source and sink for N_2_O, it showed the benefit of eliciting a reduction of N_2_O emission for an extended period after soil amendment. An interesting possibility and a future perspective lie in the possibility of combining PS-02 and CB-01 to secure effective elimination immediately after fertilization (CB-01) as well as providing a more long-lasting effect (PS-02).

Further, this enrichment technique is not restricted to the enrichment of NRB organisms but could be extended to any enrichment in which a generalist organism tolerant of environmental change is desired. Conceivably, this could include microorganisms for bioremediation, plant growth promotion, or even probiotic microorganisms. In the case of a bioremediation organism, multiple possible target materials such as soils or fresh or salt water could be cycled with a vector material such as digestate or other suitable material under the enrichment pressure of the pollutants targeted for bioremediation. Future research into the use of this enrichment strategy for different enrichment contexts should be explored and could provide valuable insights into the biology of generalist organisms and the traits which define them.

Concerns have been raised regarding persistent undesirable side effects of soil inoculation (41). Although this is highly relevant for plant-symbiotic bacteria, it seems less of a problem if the inoculant is nonsymbiotic: to our knowledge, all such nonsymbiotic inoculants tested have been found to go extinct or decline to very low abundance, albeit at different rates (15). Thus, the challenge seems to be to find isolates that persist long enough to have the desirable effect.

## MATERIALS AND METHODS

### Incubation and gas measurement.

All incubations were done in 120-mL serum vials sealed with butyl-rubber septa, using a robotized incubation system which monitors gas kinetics (O_2_, N_2_, N_2_O, NO, CO_2_, and CH_4_) by repeated sampling of the headspace, returning an equal volume of He each time (42, 43). Elaborated calculus routines, accounting for dilution by sampling and leakage (44) secured accurate estimates of production/consumption rates of each gas, electron flow rates to the various electron acceptors (O_2_, NO_3–_, NO_2–_, NO, N_2_O), and N mass-balance. Digestates and liquid cultures of isolates were stirred continuously (600 rpm) with a 23-mm-long Teflon-coated triangular magnet. Prior to incubation, the headspace air was replaced with He by repeated evacuation and filling with He and supplemented with pure N_2_O and/or O_2_ (43).

### Digestate and soils.

The digestate was taken from the anaerobic digester of a municipal wastewater treatment plant (WWTP) (13), with chemical characteristics given in Text S2A. Two clay loam soils were taken from a long-term liming experiment (45), one with pH_CaCl2_ = 6.6 (soil A) and one with pH_CaCl2_ = 5.5 (soil B). The NO_3_ + NO_2_ concentrations in the soils (after gamma sterilization) were 0.47 and 0.27 mg N kg^−1^ for soils A and B, respectively. Live (unsterilized) digestate and soil A were used in the initial enrichment cultures, while the substrates for subsequent enrichments (Fig. 1B) were autoclaved digestate (pH adjusted to 7.2 by titration with HCl) and γ-irradiated soil A (25.9 kGy, 12 months prior to experiments). Digestate used for aerobic growth of the isolated N_2_O-reducing bacteria before soil amendments (Fig. 1E) was autoclaved (121°C, 20 min), then aerated by pumping sterile filtered air through a stirred suspension of digestate for 36 h, and then pH-adjusted to ~7.50 to 7.75 (Text S2A) by titration with 4 M HCl. Aeration of the digestate was necessary in order to oxidize Fe^2+^ in the digestate to Fe^3+^, as otherwise, the abiotic reduction of O_2_ by Fe^2+^ obscured measurements of oxygen consumption (13).

10.1128/mbio.00788-22.2 Text S2 Supplemental materials and methods. Download Text S2, DOCX file, 0.02 MB. Copyright © 2022 Jonassen et al. 2022 Jonassen et al. https://creativecommons.org/licenses/by/4.0/ This content is distributed under the terms of the Creative Commons Attribution 4.0 International license.

### Dual enrichment culturing.

To enrich and isolate N_2_O-respiring organisms which can grow both in digestate and soil environments, we designed a dual enrichment approach, i.e., sequential batch cultures, alternating between sterile digestate and sterile soil as substrates (Fig. 1). Each batch was provided with a small dose of O_2_ to suppress obligate anaerobic organisms and to select organisms capable of rapid transition from O_2_ to N_2_O respiration. The enrichment series were started with two live (unsterilized) materials: 50 mL digestate (D-lines) (pH 7.6 ± 0.1) and 20 g soil A + 30 mL digestate (SD-lines) (pH 7.2 ± 0.1), each with 7 independent lines (A to G) (Fig. 1A). The nomenclature used throughout the text is *D_A-G_*_,_*_j_* and *SD_A-G_*_,_*_j_*, where *D*/*SD* denote the initial live (unsterilized) starting materials used, *A-G* denotes the 7 independent replicates, and *j* denotes the sequential enrichment number (1 to 7). D_0_/SD_0_ denote live material before enrichment with N_2_O. After replacing the headspace air with He, 3 mL (124.7 μmol) N_2_O and 3 mL (124.7 μmol) O_2_ were injected into the vials, which were then incubated at 20°C in the incubation system monitoring the O_2_, N_2_O, and N_2_. Additional N_2_O was injected when needed to avoid N_2_O depletion, while O_2_ was allowed to be depleted. Subsequent enrichment cultures (*j* = 2 to 7), alternating between γ-sterilized soil (45 g soil dry weight vial^−1^ + 16 mL sterile water) and autoclaved digestate (45 mL), were inoculated with ~10 weight percent of the previous enrichment, following the same experimental procedure and conditions as explained above for the live starting materials. At the completion of each enrichment, samples were taken for DNA extraction and analysis and for isolation in the final enrichment.

### Community analysis.

DNA was extracted in technical duplicates from all 7 biological replicate lines at the conclusion of all 7 enrichment steps, as well as from enrichment inoculant materials (D0 and SD0) and sterile materials (soil and digestate). DNA was extracted from 1 mL digestate slurry or 0.25 g soil using the PowerLyzer soil DNA extraction kit (Qiagen) following a modified kit protocol where bead beating for 30 s at 4.5 m s^−1^ in a MP Biomedicals FastPrep-24 (Thermo Fischer Scientific, Inc.) replaced the vortexing step in the manufacturer’s protocol. Quantitative digital droplet PCR (ddPCR) was performed in technical triplicates on pooled samples of DNA extracts from biological and technical replicates from each enrichment cycle (*j* = 1 to 7) and pooled samples of technical replicate DNA extractions from D_0_/SD_0_, autoclaved digestate, and γ-soil, respectively. The ddPCR reaction mix (QX200 ddPCR EvaGreen supermix; Bio-Rad) was prepared according to the manufacturer’s instructions using the universal primers PRK341F (5′-CCTACGGGRBGCASCAG-3′) and PRK806R (5′-GGACTACYVGGGTATCT-3′) (Eurofins Genomic) targeting the V3-V4 region of the 16S rRNA gene (46). The QX200 droplet generator (Bio-Rad) was used to generate oil droplet suspensions that were subjected to PCR with the parameters given in Text S2B. The PCR products were measured in a QX200 droplet reader (Bio-Rad), and the data were analyzed using QuantaSoft Analysis Pro 1.0.596 software (Bio-Rad). Microbial community composition was determined through 16S rRNA gene amplicon sequencing (V3-V4 region) and taxonomic classification of 16S rRNA gene sequences. Library preparation and sequencing data processing were performed according to Nilsen et al. (47) except that the library was quantified with the KAPA library quantification kit (Universal; Roche) in a CFX96 Touch real-time PCR detection system (Bio-Rad, USA). The amplicon library was diluted to 7 pM containing 20% PhiX before sequencing on the MiSeq platform (Illumina, USA) using the MiSeq reagent v3 kit to generate 300-bp paired-end reads. The sequencing produced 6,139,309 reads after quality filtering. The samples were rarefied at 9,000 reads, resulting in the loss of 9 samples (SD_2.1_-A, SD_7.6_-B, SD_3.7_-A, SD_7.7_-A, D_1.1_-A, D_1.3_-B, D_2.3_-A, D_2.4_-A, D_3.6_-A). The seaborn.clustermap function in the Seaborn software suite (48) was used to generate hierarchically clustered heatmaps based on Euclidian distance measures using the Ward variance minimalization linkage algorithm (49) for the 500 most abundant OTUs (sum abundance across all samples). This hierarchical clustering was used to manually assign groups of OTUs into clades on the basis of shared abundance profiles. The total 16S rRNA gene abundance (Text S5B) was combined with the relative abundance of each clade and individual OTU to provide a more quantitative assessment of organism and clade abundances throughout enrichment. The relative increases of individual OTUs from the consecutive enrichment cultures were calculated as *R_i_* = ln(*N*(*i*)/[*N*(*i*-1) · 0.1]), where N(i) is the estimated absolute abundance at the end of enrichment *i* and *N*(*i*-1) is the estimated absolute abundance at the end of the foregoing enrichment.

Principal-component analysis (PCA) was performed on a covariance matrix of OTU relative abundances using the scikit-learn software package (50) and visualized using Matplotlib (51). Similarity percentage (SIMPER) analysis (52) was performed using PAST software (53). OTU absolute abundance was calculated as the product of its relative abundance and the total abundance of 16S rRNA gene copies as assessed by ddPCR for all OTUs (16S rRNA gene copies enrichment vial^−1^).

### Isolation and characterization of N_2_O-reducing organisms.

Dilution series of the final enrichments (D_A-G_,_7_ and SD_A-G_,_7_) were spread on Sistrom’s succinate medium (SS), R2-A, tryptic soy broth (TSB), and nutrient broth (NB) agar plates (1.5 weight percent) (medium composition is given in Text S2C) and incubated anaerobically (N_2_ with ~10 volume percent N_2_O) as described in Jonassen et al. (13) (Fig. 1C). Colonies were transferred to 120-mL vials containing 50 mL of the corresponding liquid medium and incubated aerobically with stirring (700 rpm) at 20°C. 16S rRNA gene analysis showed that several different isolates were obtained, six of which were selected for full-genome sequencing: *Aeromonas* sp. AM, *Ochrobactrum* sp. OB, Pseudomonas sp. PS-02, and *Brachymonas* sp. BM, which were isolated on SS medium, and *Cloacibacterium* sp. CB-01 and CB-03, which were isolated on NB medium. Cultures were grown aerobically at 20°C in SS (AM, OB, BM, PS-02) or NB (CB-01, CB-03) medium to an optical density at 660 nm (OD_660_) of ~1.0. After centrifugation, DNA was extracted from the pellets using the PowerLyzer soil DNA extraction kit (Qiagen) as described above. The genomic DNA was sheared to approximately 8- to 14-kb-long fragments, and a library was generated with the SMRTbell Express template prep kit v2.0 (PacBio) without size selection. The library was sequenced on a PacBio SMRT cell with the PacBio Sequel system using 3.0 chemistry at the Helmholtz Centre, Munich, Germany. After data demultiplexing, the genomes of CB-01, CB-03, BM, and AM were assembled with the HGAP4 pipeline (SMRT Link Software, PacBio) with a seed coverage of 30 for CB-01, CB-03, and BM and a seed coverage of 22 for AM. PS-02 and OB were assembled with the Microbial Assembly pipeline (SMRT Link Software, PacBio) with seed coverage of 20 and 15, respectively. Genome quality was assessed with CheckM v1.0.18 (54). Annotation of coding genes was done with Prokka v1.14.5 (55) using default parameters. The draft genomes were functionally annotated for carbohydrate-active enzymes using the dbCAN2 meta server (56) and peptidases (MEROPS database, release 12.3) (57). Signal P v5.0 (58) was used to identify genes containing putative signal peptides as defined for Gram-negative bacteria. 16S rRNA genes recovered from annotated genomes were compared to OTU representative sequences using usearch.global with a sequence similarity cutoff of >97% to match isolated organisms to the OTUs which they are circumscribed by (59). To characterize the denitrification regulatory phenotypes (DRP) of our isolates, they were raised under strictly oxic conditions to secure the absence of any denitrification proteins and transferred to gas-tight vials with liquid medium containing 2 mM NO_3–_ and with He, O_2_, and N_2_O in the headspace. As these stirred cultures depleted the oxygen and switched to anaerobic respiration, they were monitored for O_2_, NO, N_2_O, and N_2_ in the headspace and NO_3–_ and NO_2–_ in the liquid as described in Jonassen et al. (13). NH_4_^+^ was also monitored in the liquid by taking 200-μL samples that were stored at −20°C before colorimetric analysis in LCK303 cuvettes (Hach Lange) in a DR 3900 spectrophotometer (Hach Lange).

### Evaluation of N_2_O-reducing isolates as N_2_O sinks in soil.

The soils A and B were amended with digestate pretreated in various ways: (i) live digestate (directly from the anaerobic digester), (ii) live digestate heat treated to 70°C for 2 h, (iii) autoclaved and pH-adjusted (7.75) digestate, (iv) autoclaved, aerated, and pH adjusted (7.75) digestate in which the isolates (AM, BM, PS-02, CB-01, or OB) had been grown by aerobic respiration, and (v) like treatment iv, with CB-01, then heated to 70°C (2 h) to kill CB-01. Each of these digestates was tested in duplicate 120-mL vials containing 10 g soil (soil A or soil B) amended with 0.6 mL digestate (i to v) and 50 μmol NO_3–_ and 41.6 μmol O_2_ in a He atmosphere (Fig. 1E). Sterilized water was added to adjust the soil water filled pore space (WFPS) to 62% ± 1% (60). The vials were incubated at 20°C and monitored for O_2_, NO, N_2_O, and N_2_ (Fig. 1D). A follow-up experiment with the same experimental design was performed to test the dose-dependent effect of three of the isolates. The isolates were grown aerobically as in treatment iv (above), the cell density achieved was assessed by the cumulated oxygen consumption (explained in detail in Text S8F), and the cell density was adjusted to 0.3 mg cell dry weight mL^−1^ for all three isolates by dilution with autoclaved digestate. These enriched digestates were then used in an amendment experiment identical to that used in treatment iv above, but with three different doses of enriched digestates (0.6, 0.3, or 0.15 mL; triplicates for each level), which is equivalent to an inoculation intensity of 18, 9, and 4.5 μg cell dry weight g^−1^ soil or 6, 3, and 1.53 · 10^7^ cells g^−1^ soil, assuming the same dry weight per cell as Paracoccus denitrificans (3 · 10^−13^ g dry weight cell^−1^). The experiment included controls, amended with equivalent doses of sterile preaerated autoclaved digestate. Finally, we tested the persistence of the isolates in soil by making an identical extra set of vials (i to v above) which were stored aerobically in moist chambers for 31 days, then amended with 1 mg ground plant material (clover) per gram of soil to secure high metabolic activity, and incubated as described above.

To assess the effect of isolates on the potential N_2_O emission from denitrification in soil, we used the N_2_O index, *I_N2O_* (30), which is the integral of the N_2_O curve divided by the integral of the total N gas, for a given period (0-*T*):

$$I_{N_{2}O}=\frac{\int_{0}^{T}N_{2}O-N\left(t\right)dt}{\int_{0}^{T}[N_{2}O-N(t)\;+N_{2}-N\left(t\right)+\mathrm{NO}(t)]\mathrm{dt}\;}$$

The time period (*T*) is not fixed but is set as the time when a given percentage of the available nitrogen oxyanions (NO_3–_ + NO_2–_) is reduced to N gas (N_2_ + N_2_O + NO). In our case, we calculated *I_N2O_* for 40% and 100% recovery of nitrogen oxyanions as N_2_ + N_2_O + NO (coined *I_N2O40%_* and *I_N2O100%_*, respectively).

### Data availability.

The sequencing data for this study have been deposited in the European Nucleotide Archive (ENA) at EMBL-EBI under accession number PRJEB44171).
