# Supplementary material for: A Dual Enrichment Strategy Provides Soil- and Digestate-Competent Nitrous Oxide-Respiring Bacteria for Mitigating Climate Forcing in Agriculture
Source: mBio. 2022 May 31;13(3):e00788-22. doi: 10.1128/mbio.00788-22 (PMC9239227; doi:10.1128/mbio.00788-22)
Supplement: Text S1 [file mbio.00788-22-s0001.docx]

## Supplementary item 1: Dual enrichment, conceptual model

**Supplementary item 1: Simulation of competition between three populations through a series of enrichment cultures.** To inspect the selection of organisms depending on experimental conditions (length of incubation, fraction of one batch transferred to the next) and the properties of the organisms, we made a simple mathematical model with three conceptual types of organisms: ***D* = digestate specialist**: fast growth in digestate, gradual death in the soil ***S* = soil specialist**: fast growth in soil, gradual death in the digestate, ***G* = generalist**: growth on both substrates, but slower than the specialists’ growth in their preferred substrate. All three were assumed to compete for the same pool of carbon substrates, and the competition was implemented by assuming logistic growth for each depending on the total increase in cell density (i.e. the sum of all three populations), and first-order death rates. The differential equations for the growth and death of the three population in a single batch are:

$\frac{dN_{D}}{dt}=N_{D}*r_{D}\left( 1-\frac{N_{t}}{K} \right)-N_{D}*d_{D}$ (1)

$\frac{dN_{S}}{dt}=N_{S}*r_{S}\left( 1-\frac{N_{t}}{K} \right)-N_{S}*d_{S}$ (2)

$\frac{dN_{G}}{dt}=N_{G}*r_{G}\left( 1-\frac{N_{t}}{K} \right)-N_{G}*d_{G}$ (3)

Using ***D*** as an example to explain the variables and parameters: ***N_D_*** is the population size of D (cells mL^-1^), ***r_D_*** (h^-1^) is its maximum growth rate (high for digestate, low/zero for soil), ***N_t_*** is the summed growth of all three populations, ***K*** is the substratum’s carrying capacity, i.e. the maximum cell number that can be produced in the substratum, ***d_D_*** (h^-1^) is the first order death rate. The growth and death rates are substrate-specific: for growth in digestate, **D** has high ***r_D_*** and low (or zero) ***d_D_***, while the opposite is the case for the organism’s growth in soil: ***r_D_*** is low (or zero), ***d_D_*** is high. The model calculates ***N_t_*** by summing up the net increase of the three populations, while any decline (death) is not affecting ***N_t_***.

The simulated cell abundances for 8 sequential enrichment batches, each lasting 100 h, and with 10% transfer from one batch to the next are shown. Parameter values were: Initial cell numbers: *N_D_* =*N_S_*= 3*10^6^, *N_G_*=3*10^3^, the maximum growth rates (h^-1^): ***r_D-dig_***= 0.15, ***r_D-soil_***=0, ***r_S-dig_***=0, ***r_S-soil_***=0.15, ***r_G-dig_***= ***r_G-soil_***=0.075, death rates were 0 except *d_D-soil_*=*d_S-dig_*=0.04, and the carrying capacity of both substrata K=10^9^ cells mL^-1^. Top panel shows abundance on log scale, bottom panel on a linear scale. D and S are sustained at stable levels (but fluctuating with substrates) only until G-abundance becomes dominant. Thereafter they decline and approach extinction if continuing the enrichment through 6-7 more batches (result not shown). The dashed line is the predicted dilution of a population which neither grows nor dies.
