# Supplementary material for: A Dual Enrichment Strategy Provides Soil- and Digestate-Competent Nitrous Oxide-Respiring Bacteria for Mitigating Climate Forcing in Agriculture
Source: mBio. 2022 May 31;13(3):e00788-22. doi: 10.1128/mbio.00788-22 (PMC9239227; doi:10.1128/mbio.00788-22)
Supplement: Text S3 [file mbio.00788-22-s0003.docx]

## Supplementary Item 3: Gas kinetics during enrichment culturing

**Supplementary Item 3A: Enrichment culturing starting with live digestate (D-line).** Panel A&B shows the result for the initial enrichment culturing by anaerobic incubation of live digestate. Panel A shows N_2_-N production rates, cumulative N_2_-N produced, and liquid concentration of N_2_O-N and O_2_ throughout enrichment, while panel B shows the measured N_2_ production rate on a log scale, together with a fitted model assuming a dying and a growing population as developed by (Jonassen et al. 2021). Panels C-H shows the same data as in panel A, for the subsequent enrichment cultures line D_A-G.2_ to D_A-G.7_. Each enrichment culture was started by transferring 10 weight % of material from the previous enriched culture (D_A-G.j_ to D_A-G.j+1_). Black arrows: exogenous addition of N_2_O. Error bars display standard deviation (n = 7).

**Supplementary Item 3B: Enrichment culturing starting with live digestate + live soil (SD-line).** Panel A&B shows the result for the initial enrichment culturing by anaerobic incubation of live digestate mixed with live soil. Panel A shows N_2_-N production rates, cumulative N_2_-N produced and liquid concentration of N_2_O-N and O_2_ throughout enrichment, while panel B shows the measured N_2_ production rate on a log scale. In contrast to the D line (**Supplementary Item 3A**), the N_2_ production increased exponentially from the very start. Panels C-H show the same data as in panel A, for the subsequent enrichment cultures line SD_A-G.2_ to SD_A-G.7_. Each enrichment culture was started by transferring 10 weight % of material from previous enriched culture (SD_A-G.j_ to SD_A-G.j+1_). Black arrows: exogenous addition of N_2_O. Error bars display standard deviation (n = 7).

**Supplementary Item 3C: Estimation of cells surviving the passage between digestate and soil and *vice versa.***

The N_2_O reduction kinetics were used to obtain estimates of the fraction of organisms surviving the transfer from one substrate to the next (from soil to digestate, and *vice versa*). The calculation was based on the assumption that all active organisms have equal growth yield (***Y*** = cells mol^-1^electrons) and maximum growth rate (***µ_max_***, h^-1^), hence also cell-specific maximum respiration rate (***v_max_***, mol electrons cell^-1^ h^-1^). The estimated number of N_2_O-respiring respiring cells at the end of enrichment ***n***, $N_{n(end)}$ is:

$N_{n(end)}=\frac{V_{e_{n(0)}}}{v_{max}}+E_{n-cum}\cdot Y$ (1)

where $V_{e_{n(0)}}$is the initial rate of electron transport to N_2_O reductase for enrichment ***n*** , and $E_{n-cum}$ is the cumulated electron flow to N_2_O during enrichment ***n***.

The estimated number of N_2_O-respiring respiring cells at the beginning of the next enrichment, ***n+1***, $N_{n+1(0)}$, is:

$N_{n+1(0)}=V_{e_{n+1}(0)}/v_{max}$ (2)

where $V_{e_{n+1}(0)}$ is initial rate of electron transport to N_2_O reductase for enrichment ***n+1***.

Since 10 % of the material in culture n was transferred to culture n+1, we have that the estimated fraction of N_2_O-reducing organisms surviving this transfer, ***f***, is:

$f=N_{n+1(0)}/(0.1\cdot N_{n(end)})$ (3)

Combining equation 1,2 and 3, and the fact that *Y ·* *v_max_* = *µ_max_*, we have that

$f=10\cdot V_{e_{n+1(0)}}/(V_{e_{n}(end)} +E_{n-cum}\cdot\mu_{max})$ (4)

While *E_n-cum_* was measured for each enrichment culture, while *V_en(0)_* and *V_en+1(0)_* were not, since each enrichment was initiated with O_2_ in the headspace, resulting in a mixture of aerobic and anaerobic respiration during the first 10-15 hours until O_2_ was depleted. To estimate *V_en(0)_* and *V_en+1(0)_* to be used to estimate ***f*** (equation 4), the measured rates of electron flow rate to N_2_O immediately after oxygen depletion were extrapolated back to time 0, assuming exponential growth rate *µ_max_* = 0.1h^-1^. The implicit assumption is the absence of any lag phase after transfer, thus $V_{e_{n+1(0)}}$ (hence ***f***) should be considered minimum estimates.

Panel A shows the principle, panel B shows a real case: the measured oxygen concentration (µM in the liquid), and the electron flow rates to terminal oxidases, to N_2_O reductase, and the total electron flow rate, based on the measured O_2_ and N_2_ production (the latter is not shown). The results are from a single vial of the last enrichment (in digestate) in the D-line.

**References**

Jonassen, Kjell Rune, Live H Hagen, Silas HW Vick, Magnus Ø Arntzen, Vincent GH Eijsink, Åsa Frostegård, Pawel Lycus, Lars Molstad, Phillip B Pope, and Lars R Bakken. 2021. 'Nitrous oxide respiring bacteria in biogas digestates for reduced agricultural emissions', *The ISME journal*: 1-11.
