# Supplementary material for: A Dual Enrichment Strategy Provides Soil- and Digestate-Competent Nitrous Oxide-Respiring Bacteria for Mitigating Climate Forcing in Agriculture
Source: mBio. 2022 May 31;13(3):e00788-22. doi: 10.1128/mbio.00788-22 (PMC9239227; doi:10.1128/mbio.00788-22)
Supplement: Text S4 [file mbio.00788-22-s0004.docx]

## Supplementary Item 4: SIMPER analysis on 16S rRNA gene amplicon data

**Supplementary Item 4A**: SIMPER analysis results output of the top 10 OTUs contribution to the explained variance in the D lines.

| Taxon | Clade | Avgerage dissimil. | Contrib. (%) | Cumulat. (%) | Mean abundance (%) | | | | | | |
| --- | --- | --- | --- | --- | --- | --- | --- | --- | --- | --- | --- |
|  |  |  |  |  | SD_A-G.1_ | SD_A-G.2_ | SD_A-G.3_ | SD_A-G.4_ | SD_A-G.5_ | SD_A-G.6_ | SD_A-G.7_ |
| OTU1 | A | 0.0659 | 48.5 | 48.5 | 0.228 | 0.155 | 0.127 | 4.96 | 7.58 | 41.4 | 32.9 |
| OTU3 | C | 0.0196 | 14.4 | 62.9 | 26.1 | 9.19 | 0.20 | 0.074 | 0.017 | 0.009 | 0.015 |
| OTU6 | A | 0.0131 | 9.64 | 72.6 | 0.104 | 6.29 | 21.9 | 20.0 | 13.9 | 8.27 | 8.36 |
| OTU2 | A | 0.0116 | 8.56 | 81.1 | 8.56 | 18.8 | 28.6 | 15.8 | 22.5 | 7.87 | 17.1 |
| OTU4 | C | 0.0064 | 4.70 | 85.8 | 15.0 | 5.08 | 0.229 | 0.054 | 0.011 | 0.007 | 0.008 |
| OTU5 | A | 0.0046 | 3.35 | 89.2 | 0.059 | 0.124 | 7.30 | 4.78 | 13.2 | 3.52 | 7.35 |
| OTU8 | A | 0.0038 | 2.78 | 92.0 | 0.039 | 0.031 | 0.014 | 2.32 | 2.91 | 6.58 | 6.98 |
| OTU14 | A | 0.0014 | 1.01 | 93.0 | 0.035 | 0.34 | 5.05 | 1.20 | 5.17 | 0.60 | 4.20 |
| OTU7 | C | 0.0010 | 0.72 | 93.7 | 5.28 | 3.53 | 0.067 | 0.023 | 0.004 | 0.004 | 0.003 |
| OTU29 | A | 0.0009 | 0.68 | 94.4 | 0.023 | 1.72 | 1.5 | 5.39 | 2.01 | 4.63 | 1.23 |

**Supplementary Item 4B**: SIMPER analysis results output of the top 10 OTUs contribution to the explained variance in the SD lines.

| Taxon | Clade | Avgerage dissimil. | Contrib. (%) | Cumulat. (%) | Mean abundance (%) | | | | | | |
| --- | --- | --- | --- | --- | --- | --- | --- | --- | --- | --- | --- |
|  |  |  |  |  | SD_A-G.1_ | SD_A-G.2_ | SD_A-G.3_ | SD_A-G.4_ | SD_A-G.5_ | SD_A-G.6_ | SD_A-G.7_ |
| OTU1 | A | 0.1033 | 61.9 | 61.9 | 0.14 | 4.2 | 14.8 | 47.0 | 43.5 | 55.3 | 39.3 |
| OTU11 | A | 0.0133 | 7.98 | 69.9 | 2.30 | 11.2 | 4.94 | 1.46 | 2.85 | 2.19 | 11.7 |
| OTU2 | A | 0.0105 | 6.31 | 76.2 | 0.36 | 2.86 | 20.7 | 5.13 | 13.4 | 3.98 | 8.91 |
| OTU6 | A | 0.0061 | 3.63 | 79.9 | 0.05 | 4.66 | 17.7 | 7.71 | 8.11 | 4.32 | 6.0 |
| OTU3 | C | 0.0057 | 3.42 | 83.3 | 14.10 | 4.61 | 0.134 | 0.023 | 0.014 | 0.010 | 0.008 |
| OTU4 | C | 0.0053 | 3.16 | 86.4 | 13.30 | 4.2 | 0.205 | 0.020 | 0.008 | 0.006 | 0.003 |
| OTU17 | A | 0.0040 | 2.37 | 88.8 | 0.25 | 12.1 | 2.21 | 2.15 | 0.321 | 0.441 | 0.106 |
| OTU7 | C | 0.0031 | 1.87 | 90.7 | 10.30 | 4.08 | 0.156 | 0.014 | 0.004 | 0.004 | 0.002 |
| OTU5 | A | 0.0029 | 1.72 | 92.4 | 0.03 | 0.469 | 10.7 | 2.62 | 5.93 | 1.82 | 4.24 |
| OTU8 | A | 0.0019 | 1.14 | 93.5 | 0.01 | 0.118 | 0.694 | 3.76 | 4.17 | 6.05 | 6.02 |
