# Supplementary material for: A Dual Enrichment Strategy Provides Soil- and Digestate-Competent Nitrous Oxide-Respiring Bacteria for Mitigating Climate Forcing in Agriculture
Source: mBio. 2022 May 31;13(3):e00788-22. doi: 10.1128/mbio.00788-22 (PMC9239227; doi:10.1128/mbio.00788-22)
Supplement: Text S5 [file mbio.00788-22-s0005.docx]

## Supplementary Item 5: Genome sequencing, phylogeny and eco-physiological genome analysis of isolated organisms

**Supplementary Item 5A**: Genome assembly statistics and parameters for isolate genomes.

| Isolates: | CB-01 | CB-03 | BM | AM | PS-02 | OB |
| --- | --- | --- | --- | --- | --- | --- |
| Assembly Parameters | | | | | | |
| Method | HGAP4 | HGAP4 | HGAP4 | HGAP4 | Microbial Assembly | Microbial Assembly |
| Seed Coverage | 30 | 30 | 30 | 22 | 20 | 15 |
| Expected Genome Length | 2 740 000 | 2 740 000 | 2 710 000 | 4 630 000 | 4 340 000 | 4 830 000 |
| Alignment to Draft Assembly | | | | | | |
| Percent Aligned Bases | 95.41% | 92.53% | 95.66% | 84.55 % | 93.60 % | 76.39 % |
| Number of Subreads (aligned) | 202 702 | 304 312 | 456 704 | 226 629 | 166 555 | 192 289 |
| Number of Polymerase Reads (aligned) | 13 088 | 20 485 | 43 392 | 18 713 | 14 278 | 14 180 |
| Polymerase Read Length Mean (aligned) [bp] | 50 243 | 50 040 | 37 734 | 38 599 | 38 419 | 42 567 |
| Polymerase Read Length Max (aligned) | 134 672 | 130 398 | 121 149 | 122 221 | 125 758 | 123 538 |
| Polished Assembly | | | | | | |
| Polished Contigs | 1 | 1 | 2 | 1 | 5 | 54 |
| Maximum Contig Length [bp] | 2 979 886 | 2 718 917 | 2 711 532 | 4 571 002 | 4 016 625 | 297 087 |
| Sum of Contig Lengths [bp] | 2 979 886 | 2 718 917 | 2 754 828 | 4 571 002 | 4 494 782 | 4 640 821 |
| Coverage | | | | | | |
| Mean Coverage | 207 | 354 | 552 | 146 | 113 | 120 |
| Missing bases (%) | 0.00 % | 0.00 % | 0.00 % | 0.00 % | 0.00 % | 0.00 % |
| CheckM quality parameters | | | | | | |
| Completeness | 100 % | 100 % | 99.77 % | 99.97 % | 99.96 % | 80.32 % |
| Contamination | 0.74 % | 0.25 % | 0 % | 0 % | 1.16 % | 0.43 % |

**Supplementary Item 5B:** ddPCR quantification of 16S rRNA gene copy numbers on pooled samples (A to G) of DNA extracts from D_A-G.j_ and SD_A-G.j_ (j = 1-7), D0 and SD0, and sterile growth substrates used throughout the enrichment (AC-dig and ϒ-Soil) and standard error (n = 3).

| **Line** | **Material:** | **Sample:** | **16S/vial:** | **SE** |
| --- | --- | --- | --- | --- |
| D | Live digestate | D_0_ | 3.50E+11 | 7.0E+09 |
|  | Live digestate | D_A-G.1_ | 1.80E+11 | 4.7E+09 |
|  | ϒ Soil | D_A-G.2_ | 1.40E+11 | 7.9E+09 |
|  | AC-Dig | D_A-G.3_ | 1.60E+11 | 4.4E+09 |
|  | ϒ Soil | D_A-G.4_ | 2.10E+11 | 1.4E+09 |
|  | AC-Dig | D_A-G.5_ | 2.00E+11 | 7.1E+09 |
|  | ϒ Soil | D_A-G.6_ | 2.50E+11 | 7.4E+09 |
|  | AC-Dig | D_A-G.7_ | 2.00E+11 | 6.3E+09 |
| SD | Live dig:soil mix | SD_0_ | 2.60E+11 | 2.1E+09 |
|  | Soil:mix after enr. | SD_A-G.1_ | 1.60E+11 | 9.9E+09 |
|  | ϒ Soil | SD_A-G.2_ | 1.10E+11 | 6.9E+09 |
|  | AC-Dig | SD_A-G.3_ | 1.10E+11 | 5.0E+09 |
|  | ϒ Soil | SD_A-G.4_ | 2.60E+11 | 9.7E+09 |
|  | AC-Dig | SD_A-G.5_ | 1.70E+11 | 7.3E+09 |
|  | ϒ Soil | SD_A-G.6_ | 2.70E+11 | 3.0E+09 |
|  | AC-Dig | SD_A-G.7_ | 1.90E+11 | 4.0E+09 |
| Growth substrate | AC-Dig | Growth substrate | 7.00E+10 | 6.8E+09 |
|  | ϒ Soil | Growth substrate | 1.60E+10 | 1.8E+08 |


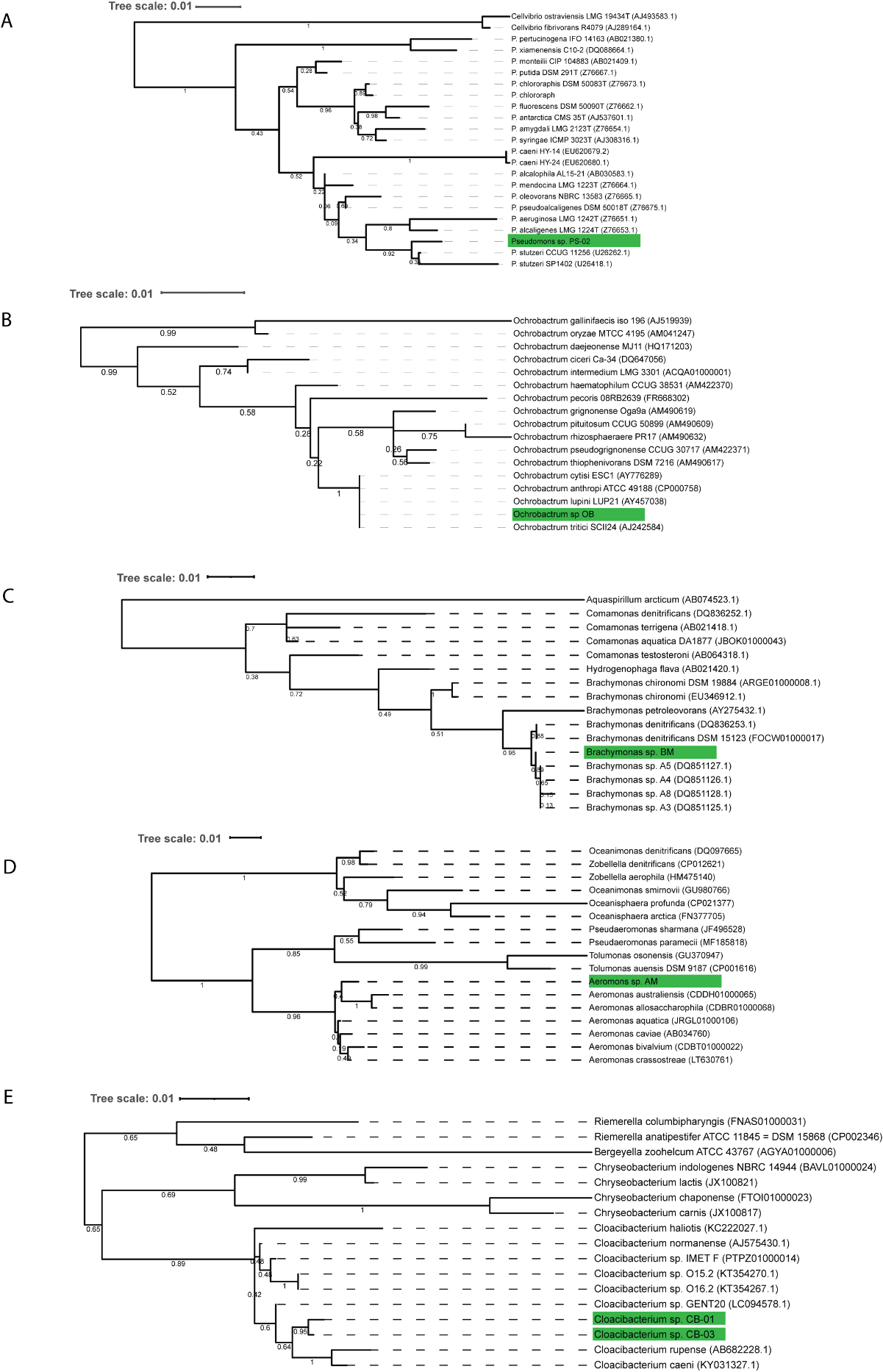


**Supplementary Item 5C**: Neighbor joining tree (100 bootstrap samplings) derived from alignment of 16S rRNA gene sequences from isolates and neighboring organisms. Numbers represent the percentage of bootstrap samplings that generate at each node. Species names are followed by the accession numbers of their 16S rRNA gene sequences. Panel A: *Pseudomonas* sp. PS-02, Panel B: *Ochrobactrum* sp. OB, Panel C: *Brachymonas* sp. BM, Panel D: *Aeromonas* sp. AM and Panel E: *Cloacibacterium* sp. CB-01 and CB-03.

**Supplementary Item 5D:** The predicted carbohydrate-active enzymes (CAZymes) in the genomes of AM, BM, CB-01, CB-03, PS-02, and OB. The CAZymes were automatically annotated through the dbCAN meta server (Feb 2021) using HMMER, DIAMOND and Hotpep, as well as including SignalP annotation of signal peptides. The CAZyme assignments include the enzyme classes Glycoside Hydrolysis (GHs), Glycosyl transferases (GTs), Polysaccharide lyases (PLs), Carbohydrate Esterases (CEs) and enzymes with Auxiliary Activity (AAs) in addition to Carbohydrate-Binding Modules (CBM). Identical annotation in ≥ 2 tools was required for a CAZY assignment to be considered robust. Prokka annotations of corresponding genes is given in **Supplementary Data S1**.

**Supplementary Item 5E:** Identified enzymes predicted as carbohydrate-active enzymes (CAZymes) from the dbCAN meta-server with corresponding PROKKA annotations of enzymes targeting extracellular carbohydrates (indicated by identified signal sequence for membrane trans allocation (Signal P)) as well as genes encoding proteins involved in glycogen metabolism in the genomes of PS-02, OB, BM, AM, CB-01 and CB-03. BM did not contain annotated CAZymes of particular relevance. A complete list of dbCAN grouped genes is given together with corresponding Prokka annotations for each genome in **Supplementary Data S1.**

**Supplementary Item 5F:** MEROPS annotated peptidases in the genomes of PS-02, OB, BM, AM, CB-01 and CB-03. Every protein sequence was screened for presence of signal peptide sequences in SignalP 5.0 and the MEROPS subfamilies were collapsed to families. Prokka annotations of corresponding genes is given in **Supplementary Data S1**
