# Supplementary material for: A Dual Enrichment Strategy Provides Soil- and Digestate-Competent Nitrous Oxide-Respiring Bacteria for Mitigating Climate Forcing in Agriculture
Source: mBio. 2022 May 31;13(3):e00788-22. doi: 10.1128/mbio.00788-22 (PMC9239227; doi:10.1128/mbio.00788-22)
Supplement: Text S6 [file mbio.00788-22-s0006.docx]

## Supplementary Item 6: Assessment of growth or death of OTU’s

 **Supplementary Item 6A: Assessment of growth/death (soil versus digestate) of OTU’s within Clade A**. To assess growth or decline of each OTU within the 6 clades, we calculated the relative increase for each consecutive enrichment culture as *R*= ln(_N(i)_/(N_(i-1)_*0.1) where N_i_ is the estimated copy number per vial at the end of enrichment i and N_(i-1)_ is the estimated copy number per vial at the end of the foregoing enrichment (both estimated by the relative abundance of the OTU in question and the total copy numbers of 16S rRNA gene quantified by digital droplet PCR (**Supplementary Item 5B**). The multiplication with 0.1 is because 10% of the content of one enrichment culture was transferred to the next. *R* for the initial enrichment in live digestate (with and without live soil added is ln(N/N_0_), where N is the abundance after enrichment and N_0_ is the abundance measured at the onset of this enrichment). This figure shows the results for OTU’s within Clade A: *R_soil_* plotted against *R_digestate_*, with standard error (n=8) marked by vertical and horizontal lines. The dashed line is the plotted equation *R_soil_*+*R_digestate_*= 4.6, which marks a division between OTU’s which increase (R_soil_+R_digestate_ > 4.61) and those that are gradually washed out (R_soil_+R_digestate_ < 4.61) throughout the dual enrichment cultures. The plotting of *R_soil_* and *R_digestate_* for individual OTU’s shows that the OTU’s within Clade A span a continuum from “Soil specialists” (high *R_soil_*, low/negative *R_digestate_* values) through “Generalists” (similar *R_soil_* and *R_digestate_* values >2) and further on to “Digestate specialists” (low R_soil_, high R _dig_). *R_soil_* was negatively correlated with *D_digestate_* (regression function: *R_soil_*=4.7-0.6·*R_digestate_*, r^2^=0.7, p<0.01).

**Supplementary Item 6B: Assessment of growth/death (soil versus digestate) of OTU’s within Clade B**. Calculus and plotting are explained in the legend of **Supplementary Item 6A**. All OTU’s within Clade B were gradually diluted out (below the dashed line).

**Supplementary Item 6C: Assessment of growth/death (soil versus digestate) of OTU’s within Clade C**. Calculus and plotting are explained in the legend of **Supplementary Item 6A**. All OTU’s within Clade B were diluted out (below the dashed line).

**Supplementary Item 6D: Assessment of growth/death (soil versus digestate) of OTU’s within Clade D**. Calculus and plotting are explained in the legend of **Supplementary Item 6A.** For digestate, the majority of OTU’s had R between -2 and -1.5, which indicates a 80-85% decline during the enrichment in digestate. In contrast, R for enrichments in soil ranged from 3-7, indicating that the abundance increased by a factor of 150-1100 (7-10 cell divisions) during the enrichment in soil. A clear negative correlation between R_soil_ and R_digestate_ is observed (r^2^=0.573, p<0.01). The outliers with apparent growth in digestate (R>0) showed somewhat erratic development of abundance throughout, as illustrated for OTU1369 (inset panel). The majority of the OTU’s are below the line (declining), while some are sustained or increase slightly, which was the case for OTU74 circumscribing the isolated *Ochrobactrum* sp. OB. The distribution of R values suggests that the majority dies out fast in the digestate but grow fast in soil.

**Supplementary Item 6E: Assessment of growth and death of OTU’s within Clade E**. Calculus and plotting are explained in the legend of **Supplementary Item 6A.** 35 of the 50 OTU’s within this clade are above the line, hence not diluted out, although they did not reach dominance.

**Supplementary Item 6F: Assessment of growth and death of OTU’s within Clade F**. Calculus and plotting are explained in the legend of **Supplementary Item 6A.** 46 of the 57 OTU’s within this clade are above the line, indicating that they were sustained throughout the enrichment, but none reached dominance.
