# Supplementary material for: A Dual Enrichment Strategy Provides Soil- and Digestate-Competent Nitrous Oxide-Respiring Bacteria for Mitigating Climate Forcing in Agriculture
Source: mBio. 2022 May 31;13(3):e00788-22. doi: 10.1128/mbio.00788-22 (PMC9239227; doi:10.1128/mbio.00788-22)
Supplement: Text S8 [file mbio.00788-22-s0008.docx]

## Supplementary Item 8: Growth in autoclaved digestate and effects on soil emissions

**Supplementary Item 8A:** **Aerobic growth in autoclaved digestate.** AM, OB, BM, PS-02 and CB-01 were raised aerobically in 50 mL SS (AM, OB, BM and PS-02) or 50 mL NB (CB-01) to high cell densities (OD_660nm_ ~ 1), then transferred (1 mL) to vials with 50 mL stirred (600 rpm) autoclaved pH-adjusted (pH=7.75) and pre-aerated (aerated by pumping sterile filtered air through a stirred suspension for 36 hours) digestate at 20 °C. Panels A-G show oxygen concentration (red), arrows (exogenous O_2_ additions), cumulative O_2_ reduced (blue), rate of oxygen consumption (green). Aeration of the autoclaved digestate was necessary to secure near-complete abiotic oxidation of the Fe^2+^ in the digestate, which would otherwise obscure measurements of O_2_ consumption. Panel A: *Aeromonas sp*. AM (n=3). Panel B: Pseudomonas sp. PS-02 (n=2). Panel C: *Ochrobactrum sp*. OB (n=3). Panel D: *Cloacibacterium* sp. CB (n=3). Panel E: *Brachymonas sp*. BM (n=3), Panel G: Non-aerated, pH adjusted (pH = 7.65) autoclaved digestate (n = 5). Panel F: Control: Aerated, pH adjusted (pH = 7.75) autoclaved digestate (n = 5). Panel H: The cumulated oxygen consumption by each strain used to estimate the number of cells produced, assuming that the growth yield for all strains is the same as for *Paracocus denitrificans*, which is 30 g cell dry-weight mol^-1^ O_2_ (based on: 2·10^-13^ g dry-weight per cell, growth yield = 1.5·10^14^ cells mol^-1^ O_2_ (Bergaust et al. 2010)). Panel H shows estimated amount of cell dry-matter mL^-1^ for each strain (left y-axis) with the number of cells mL^-1^ indicated below the x-axis labels. The number of genes coding for glycosyl hydrolases (**GH**) and proteases (**P**) in the genome of each strain (from **Supplementary Item 5D-F**) is shown (right y-axis). **P** and **GH** were correlated (r^2^=0.93), and the cell dry-weight was correlated to both (r^2^= 0.97 for both).

**Supplementary Item 8B**: **Incubation of digestate enriched with isolates (Supplementary Item 8A) (0.6 mL), live digestate (0.6 mL) and heat treated digestate (0.6 mL) in soil with pH=5.5 (10 g) at 20 °C.** Panel A: kinetics of O_2_, NO, N_2_O and N_2_ throughout the incubation of soils amended with the various materials (one panel for each amendment). Average values shown (n=2). Initial oxygen (~40 µmol vial^-1^) corresponds to ~1.0 vol% in the headspace. The amounts of O_2_, NO and N_2_O are as measured, while “Cumulative N_2_-N” denotes the measured N_2_ that is corrected for leakage and losses by sampling (see (Molstad, Dörsch, and Bakken 2007)). The N_2_ and N_2_O kinetics were used to calculate the N_2_O index (***I_N2O_***), which is the area under the N_2_O curve divided by the area under the N_2_O+N_2_ curve for a specific time span. ***I_N2O_*** values are shown in **Fig 5** in the main paper and is a proxy for the propensity of denitrification to emit N_2_O. Panel B: peak (maximum) amounts of NO and N_2_O (results for single vials). NO is shown as nM in the liquid phase (equilibrium concentrations with measured NO in headspace), while N_2_O is shown as µmol N_2_O- N vial^-1^.

**Supplementary Item 8C**: **Incubation of digestate enriched with isolates (Supplementary Item 8A) (0.6 mL), live digestate (0.6 mL) and heat treated digestate (0.6 mL) in soil with pH=6.6 (10 g) at 20 °C.** Panel A: kinetics of O_2_, NO, N_2_O and N_2_ throughout the incubation of soils amended with the various materials (one panel for each amendment). Average values shown (n=2). Initial oxygen (~40 µmol vial^-1^) corresponds to ~1.0 vol% in the headspace. The amounts of O_2_, NO and N_2_O are as measured, while “Cumulative N_2_-N” denotes the measured N_2_ that is corrected for leakage and losses by sampling (see (Molstad, Dörsch, and Bakken 2007)). The N_2_ and N_2_O kinetics were used to calculate the N_2_O index (***I_N2O_***), which is the area under the N_2_O curve divided by the area under the N_2_O+N_2_ curve for a specific time span. ***I_N2O_*** values are shown in **Fig. 5** in the main paper and is a proxy for the propensity of denitrification to emit N_2_O. Panel B: peak (maximum) amounts of NO and N_2_O (results for single vials). NO is shown as nM in the liquid phase (equilibrium concentrations with measured NO in headspace), while N_2_O is shown as µmol N_2_O- N vial^-1^.

**Supplementary Item 8D**: **Incubation of digestate enriched with isolates (Supplementary Item 8A) (0.6 mL), live digestate (0.6 mL) and heat treated digestate (0.6 mL) in soil with pH=5.5 (10 g) at 20 °C after aerobic storage for 1 month (30 days) under oxic conditions (20 °C).** Panel A: kinetics of O_2_, NO, N_2_O and N_2_ throughout the incubation of soils amended with the various materials (one panel for each amendment). Average values shown (n=2). Initial oxygen (~40 µmol vial^-1^) corresponds to ~1.0 vol% in the headspace. The amounts of O_2_, NO and N_2_O are as measured, while “Cumulative N_2_-N” denotes the measured N_2_ that is corrected for leakage and losses by sampling (see (Molstad, Dörsch, and Bakken 2007)). The N_2_O index (***I_N2O_***), which is the area under the N_2_O curve divided by the area under the N_2_O+N_2_ curve for a specific time span, was not calculable for most treatments as the experiment was not run until all available oxyanions was reduced to N_2_ or N_2_O (increasing Cumulative N_2_-N for most vials). Panel B: peak (maximum) amounts of NO and N_2_O (results for single vials). NO is shown as nM in the liquid phase (equilibrium concentrations with measured NO in headspace), while N_2_O is shown as µmol N_2_O-N vial^-1^.

**Supplementary Item 8E**: **Incubation of digestate enriched with isolates (Supplementary Item 8A) (0.6 mL), live digestate (0.6 mL) and heat treated digestate (0.6 mL) in soil with pH=6.6 (10 g) at 20 °C were done after storage for 31 days under oxic conditions (20°C).**  Panel A: kinetics of O_2_, NO, N_2_O and N_2_ throughout the incubation of soils amended with the various materials (one panel for each amendment). Average values shown (n=2). Initial oxygen (~40 µmol vial^-1^) corresponds to ~1.0 vol% in the headspace. The amounts of O_2_, NO and N_2_O are as measured, while “Cumulative N_2_-N” denotes the measured N_2_ that is corrected for leakage and losses by sampling (see (Molstad, Dörsch, and Bakken 2007)). The N_2_O index (***I_N2O_***), which is the area under the N_2_O curve divided by the area under the N_2_O+N_2_ curve for a specific time span, was not calculable for most treatments as the experiment was not run until all available oxyanions was reduced to N_2_ or N_2_O (increasing Cumulative N_2_-N for most vials). The green box indicates isolates PS-02 and CB-01. Panel B: peak (maximum) amounts of NO and N_2_O (results for single vials). NO is shown as nM in the liquid phase (equilibrium concentrations with measured NO in headspace), while N_2_O is shown as µmol N_2_O- N vial^-1^. While PS-02 had a statistically significant effect on maximum N_2_O, the apparent effect of CB-01 was not statistically significant.

**Supplementary Item 8F:** **Aerobic growth of isolated organisms in autoclaved digestate for dose response experiment.** The autoclaved digestate to be used for cultivation was pH adjusted to 7.6 and vigorously aerated (sparging for 36 hours) before use. The aeration was necessary because previous experiments had demonstrated substantial abiotic O_2_-consumption by oxidation of Fe^2+^ in autoclaved digestate which would obscure the measurement of aerobic respiration by the bacteria (see **Supplementary Item 8A**). Pre-cultures of CB-01, PS-02 and OB were grown aerobically in NB medium (CB-01) and SS medium (PS-02 and OB) to OD_660nm_ 0.798, 0.379 and 0.786, respectively, and used to inoculate 120 mL vials (1 mL per vial) containing 50 mL digestate (with a magnetic bar), which were capped (butyl rubber septa), incubated at 20 ^o^C with vigorous stirring (600 rpm), and monitored for O_2_ concentration in the headspace. When needed, to secure oxic conditions, more O_2_ was injected. Panels A – D: Oxygen concentration (red), O_2_ injection (black arrows), cumulative O_2_ reduction (blue) rate of oxygen consumption (green). Panel A: *Cloacibacterium sp*. CB-01 (n=3). Panel B: *Pseudomonas* sp. PS-02 (n=3). Panel C: *Ochrobactrum sp*. OB (n=3). Panel D: Control: no bacteria (n=3). The cumulated oxygen consumption by each strain was used to estimate the number of cells produced, assuming that the growth yield for all strains is the same as for *Paracocus denitrificans*, which is 30 g cell dry-weight mol^-1^ O_2_ (based on (Bergaust, Bakken, and Frostegård 2010): 2·10^-13^ g dry-weight per cell, growth yield= 1.5·10^14^ cells mol^-1^ O_2_). The estimated amount of cell dry-weight for the three strains was 0.36 (±0.01), 0.67 (±0.04) and 0.74 (± 0.02) mg cell dry-weight mL^-1^ for CB-01, PS-02 and OB, respectively. Assuming that the three strains have the same amount of dry-weight per cell as *Paracoccus* (2·10^-13^ g cell^-1^) the estimated number of “Paracoccus equivalents” are 1.8, 3.4 and 1.9 ·10^9^ cells mL^-1^ for CB-01, PS-02 and OB, respectively.

**Supplementary Item 8G**: **Incubation of digestate enriched with isolates OB, PS-02 and CB-01 and aerated, pH adjusted, autoclaved digestate (Control) in 10 g pH 6.6 soil supplemented with 25 µmol NO_3_^-^ and 0.5 mL O_2_**. Panel A: kinetics of O_2_, NO, N_2_O and N_2_ throughout the incubation of soils amended with the various materials (one panel for each amendment). Average values shown, with standard deviation (n=3). Initial oxygen (~20 µmol vial^-1^) corresponds to ~0.5 vol% in the headspace. The amounts of O_2_, NO and N_2_O are as measured, while “Cumulative N_2_-N” denotes the measured N_2_ that is corrected for leakage and losses by sampling (see (Molstad, Dörsch, and Bakken 2007)). The digestate enriched with the isolates (**Supplementary Item 8F**) was diluted with sterile aerated digestate (as used in the controls) to give the same cell concentrations per mL digestate (~2 · 10^8^ N_2_O reducing cells mL^-1^ digestate). Error bars show standard deviation (n = 3 for all treatments, besides PS-02 0.15 mL where n = 2). Panel B: average peak (maximum) amounts of NO and N_2_O. NO is shown as nM in the liquid phase (equilibrium concentrations with measured NO in headspace), while N_2_O is shown as µmol N_2_O- N vial^-1^. Two ***I_N2O_*** values are shown: one for the timespan until 40% of the NO_3_^-^ -N is recovered as N_2_+N_2_O+NO-N (**I_N2O 40%_**), and one for 100% recovery (**I_N2O 100%_**).

**Supplementary Item 8H: Summary data for dose response experiment.** The table shows the N_2_O index values calculated for the period until 40 and 100% of NO_3_ was converted to NO+N_2_O+N_2_ (*I_N2O_* 40% and *I_N2O_* 100%, respectively), and the maximum N_2_O concentration reached (Max N_2_O) for the dose experiment (**Supplementary Item 8G**). Average values with standard deviations are given for each treatment (n=3 replicate vials). Treatments are digestate with bacteria (CB-01, PS-02 and OB), digestate without bacteria (Control), and 3 doses of digestate: 0.6, 0.3 and 0.15 mL digestate vial^-1^ (containing 10 g soil). The digestates with bacteria contained 0.3 mg bacterial cell dry-weight mL^-1^, hence the inoculation intensities of the three doses were 18, 9 and 4.5 µg cell dry-weight g^-1^ soil. The third column for each variable shows the value expressed as a % of the control value at the same inoculum intensity. A significantly lower value for the bacterial treatment versus control is marked by * (p>0.05, t-test).

| **Dose** (mL vial-1) | **Strain** | ***I_N2O_* 40%** | | | ***I_N2O_* 100%** | | | **Max N_2_O (µmol N vial^-1^)** | | |
| --- | --- | --- | --- | --- | --- | --- | --- | --- | --- | --- |
|  |  | **Avg** | **St.dev** | **% of contr** | **Avg** | **St.dev** | **% of contr** | **Average** | **Stdev** | **% of contr** |
| **0.6** | CB-01 | 0.027 | 0.005 | **4 *** | 0.006 | 0.001 | **2 *** | 0.64 | 0.16 | **4 *** |
| **0.6** | PS-02 | 0.41 | 0.044 | **55 *** | 0.172 | 0.04 | **53 *** | 9.13 | 0.80 | **54 *** |
| **0.6** | OB | 0.65 | 0.012 | **88 *** | 0.239 | 0.03 | **73 -** | 10.68 | 0.53 | **63 *** |
| **0.6** | Control | 0.75 | 0.026 |  | 0.327 | 0.11 |  | 17.03 | 2.66 |  |
| **0.3** | CB-01 | 0.28 | 0.001 | **36 *** | 0.127 | 0.01 | **39 *** | 5.62 | 0.42 | **30 *** |
| **0.3** | PS-02 | 0.60 | 0.006 | **80 *** | 0.207 | 0.03 | **63 -** | 11.00 | 0.21 | **58 *** |
| **0.3** | OB | 0.59 | 0.181 | **78 -** | 0.172 | 0.01 | **52 *** | 11.34 | 0.58 | **60 *** |
| **0.3** | Control | 0.76 | 0.015 |  | 0.330 | 0.10 |  | 18.99 | 4.61 |  |
| **0.15** | CB-01 | 0.49 | 0.019 | **67 *** | 0.222 | 0.01 | **93 -** | 10.62 | 0.36 | **80 *** |
| **0.15** | PS-02 | 0.64 | 0.034 | **88 -** | 0.159 | 0.01 | **66 *** | 10.50 | 0.30 | **79 *** |
| **0.15** | OB | 0.70 | 0.010 | **97 -** | 0.152 | 0.00 | **63 *** | 10.75 | 0.35 | **81 *** |
| **0.15** | Control | 0.72 | 0.011 |  | 0.240 | 0.03 |  | 13.30 | 0.66 |  |

**References**

Bergaust, Linda, Yuejian Mao, Lars R Bakken, and Åsa Frostegård. 2010. 'Denitrification response patterns during the transition to anoxic respiration and posttranscriptional effects of suboptimal pH on nitrogen oxide reductase in Paracoccus denitrificans', *Applied and environmental microbiology*, 76: 6387-96.

Molstad, Lars, Peter Dörsch, and Lars R Bakken. 2007. 'Robotized incubation system for monitoring gases (O2, NO, N2O N2) in denitrifying cultures', *Journal of microbiological methods*, 71: 202-11.
