# Supplementary material for: A Dual Enrichment Strategy Provides Soil- and Digestate-Competent Nitrous Oxide-Respiring Bacteria for Mitigating Climate Forcing in Agriculture
Source: mBio. 2022 May 31;13(3):e00788-22. doi: 10.1128/mbio.00788-22 (PMC9239227; doi:10.1128/mbio.00788-22)
Supplement: Text S7 [file mbio.00788-22-s0007.docx]

## Supplementary Item 7: pH-dependent aerobic and anaerobic respiration by Cloacibacterium sp. CB-01

**Supplementary Item 7: pH-dependent aerobic and anaerobic respiration by *Cloacibacterium* sp. CB-01.** CB-01 was grown in gas tight 120 mL vials with 50 mL NB-medium, with pH ranging from 5.5 to 7, initially supplemented with 1 mL O_2_ and 1 mL N_2_O at constant temperature and stirring (20 °C, 600 rpm). Initial OD_660_ ≈ 0.001. Top panel**:** First period of O_2_ reduction (rate) during incubations with media adjusted to different pH levels. Bottom panel: First period of N_2_O reduction (rate) during incubations with media adjusted to different pH. Standard deviations shown as vertical error bars (n = 3).
