# Supplementary material for: A Dual Enrichment Strategy Provides Soil- and Digestate-Competent Nitrous Oxide-Respiring Bacteria for Mitigating Climate Forcing in Agriculture
Source: mBio. 2022 May 31;13(3):e00788-22. doi: 10.1128/mbio.00788-22 (PMC9239227; doi:10.1128/mbio.00788-22)
Supplement: Text S2 [file mbio.00788-22-s0002.docx]

## Supplementary Item 2: Supplementary materials and methods

### Materials for inoculum and growth substrates

**Supplementary Item 2A: Digestate characteristics at the time of sampling for enrichment culturing and soil incubations**. The digestate was taken from an anaerobic digester of a municipal WWTP (same as used by (Jonassen et al. 2021)). Enrichment culturing and soil incubations were done with freshly sampled digestate (Sampling 1-4). Digestate characteristics were analyzed in the NS-EN ISO/IEC 17025 accredited laboratory belonging to the WWTP.

|  | **Digestate characteristics** | | | | | | |
| --- | --- | --- | --- | --- | --- | --- | --- |
|  | pH | % dry weight ^a^ | LOI ^b^  (% of DW) | TAK ^c^  (meq L^-1^) | VFA ^c^  (meq L^-1^) | VFA/TAK | NH_3+_NH_4_^+^  (mg-N L^-1^) |
| Sample 1^d^ | 7.61 | 4.12 | 55.93 | 186 | 16.7 | 0.090 | nd. |
| Sample 2^d^ | 7.73 | 3.97 | 55.45 | 199 | 17.3 | 0.087 | nd. |
| Sample 3^d^ | 7.84 | 3.87 | 55.46 | 201 | 16.3 | 0.081 | nd. |
| Sample 4^d^ | 7.60 | 3.73 | 54.87 | 189 | 15.2 | 0,080 | 1486 ± 7^e^ |

^a^ Dry weight as % of wet weight. ^b^ Loss of ignition, % of dry weight. ^c^ VFA = volatile fatty acids. TAK = total alkalinity (determined by titration described in EN12176:1998, given by WWTP). ^d^ Sample 1: sample used in enrichment experiment (live digestate inoculum, D_A-G.1_ and SD_A-G.1_, shown in **Fig. 2A** in main paper and **Supplementary Item 3A**, **3B** and **3C**. Source of autoclaved digestate used as growth substrate in enrichment culturing. Sample 2 was used for aerobic growth of isolates, (**Supplementary Item 8F**). Sample 3 was used in soil incubations (live digestate) (**Fig. 5** in main paper and **Supplementary Item 8B-E**), Sample 4 was used in soil dose response experiment (**Supplementary Item 8G**). ^e^ Aeration of autoclaved digestate removed some NH_4_^+^ (concentration in digestate before aeration was 1883 mg ± 3 mg NH_4_-N L^-1^).

**Supplementary Item 2B**: PCR cycle settings for 16S rRNA gene ddPCR with primer pairs PRK341F/PRK806R.

| Time: | Temperature (°C): | Description: | |  |
| --- | --- | --- | --- | --- |
| 5 min | 95 | Denaturation | |  |
| 30 s | 95 | Denaturation | 40 cycles | |
| 30s | 55 | Annealing |  |  |
| 45 s | 72 | Extension |  |  |
| 5 min | 4 | Signal stabilization | |  |
| 5 min | 90 | Signal stabilization | |  |
| Indef. | 4 | Hold step | |  |

**Supplementary Item 2C: Media composition**

Sistrom’s succinate medium (**SS**): contained (L^-1^) 3.48 g K_2_HPO_4_, 0.195 g NH_4_Cl, 4 g succinic acid, 0.10 g glutamic acid, 0.04 g aspartic acid, 0.5 g NaCl, 0.2 g nitrilotriacetic acid, 0.3 g MgSO_4_ · 7H_2_O, 0.015 g CaCl_2_ · 7H_2_O, 0.002 g FeSO_2_ · 7H_2_O, 0.1 mL trace element solution and 0.1 mL vitamin solution. The trace element solution contained (g L^-1^): 17.65 g EDTA (triplex 3), 109.5 g ZnSO_4_ · 7H_2_O, 50 g FeSO_4_ · 7H_2_O, 15.4 g MnSO_4_ · H_2_O, 3.92 g CuSO_4_ · 5H_2_O, 2.48 g Co(NO_3_)_2_ · 6H_2_O and 1.14 g H_3_BO_3_. H_2_SO_4_ was added until the solution cleared. The vitamin solution contained (g L^-1^) 10.0 g nicotinic acid, 5.0 g thiamine HCl and 0.10 g Biotin. Solid media agar plates were produced by addition of 1.5 wt.% agar. R-2A medium (**R-2A**): contained (L^-1^) 0.5 g casein acid hydrolysate, 0.5 g dextrose, 0.3 g K₂HPO₄, 0.025 g MgSO₄, 0.5 g proteose peptone, 0.3 g sodium pyruvate 0.5 g starch (soluble), 0.5 g yeast extract. **R-2A** (Merck 17209) was used for preparing agar plates. Tryptic soy broth (**TSB**): containing (L^-1^) 17 g casein peptone, 3 g soya peptone, 5 g NaCl, 2.5 g Na_2_HPO_4_ and 2.5 g dextrose (Sigma Aldrich 22092-500G). 1.5 wt. % agar plates were made with 0.1X strength TSB. Nutrient broth (**NB,** Merck): containing (L^-1^) 15 g yeast extract, 3.0 g NaCl, 1 g dextrose. 1.5 wt.% agar plates were made with 0.2X strength NB.

**References**

Jonassen, Kjell Rune, Live H Hagen, Silas HW Vick, Magnus Ø Arntzen, Vincent GH Eijsink, Åsa Frostegård, Pawel Lycus, Lars Molstad, Phillip B Pope, and Lars R Bakken. 2021. 'Nitrous oxide respiring bacteria in biogas digestates for reduced agricultural emissions', *The ISME journal*: 1-11.
